# Brewers’ spent hop revalorization for the production of high added-value cosmetics ingredients with elastase inhibition capacity

**DOI:** 10.1038/s41598-022-26149-3

**Published:** 2022-12-21

**Authors:** Maria Paredes-Ramos, Enma Conde Piñeiro, Jose M. Lopez Vilariño

**Affiliations:** 1 Hijos de Rivera S.A.U., C/José María Rivera Corral 6, A Coruña, Spain; 2 GLECEX S.L. (Global and Ecofriendly Natural Extracts S.L.), Edificio CITI, Parque Tecnolóxico de Galicia, San Cibrao das Viñas, Ourense Spain

**Keywords:** Analytical biochemistry, Computational biology and bioinformatics, Molecular modelling, Proteases

## Abstract

This article summarizes the analysis of α and β-acids and prenylflavonoids from brewers’ spent hop (BSH) as source of bioactive molecules to improve skin integrity by inhibiting elastase activity. To maximize the efficacy of the BSH extracts, it was necessary to identify the most bioactive hop compounds and the extraction parameters to maximize elastase inhibition and total antioxidant capacity. Thus, a computational methodology was carried out to test the anti-elastase potential of these hop molecules, detecting cis-iso-α-cohumulone and 8-prenylnaringenin as main inhibitors. Then, BSH extracts were optimized to ensure the maximum extraction of bioactive compounds, using compatible solvents (water and 100% plant-based propanediol) according to the green cosmetic standards. Finally, a determination and quantification method based on HPLC–MS/MS was used to guarantee the presence of the bioactive molecules, detecting a higher concentration of cis-iso-α-cohumulone and 8-prenylnaringenin in those samples with high anti-elastase activity. By optimizing extraction conditions and agents, a BSH extract was designed, showing high antioxidant (81.9 mmol Trolox/L) and high anti-elastase capacities.

## Introduction

Industrial production of beer, which amounts up to 340 million hL for the European Union in 2020, generates around 14 kg/hL of by-products, mostly brewers’ spent grain (BSG) but also spent hop (BSH) and yeast (BSY), which can be reutilized directly for food and feed purposes or as fertilizer for soil amendment, amongst others^1–3^. These applications require low investment but are also low profitable for beer companies, so during the last years, several upcycling applications have arisen to transform BSG, BSH and BSY to high added-value products as microbial biomass for single cell protein production, to sweeteners as xylitol, utilized for low-sugar food products development, or as source of polyphenols and antioxidants for food and cosmetics industries^1,4–6^.

These high added-value applications are aligned with the Green Deal agenda of the European Union but also with the global well-being trend which encourages consumers to take care of their health and skin protection. On this regard, one of the aspects which is being considered at increasingly younger ages is skin ageing. The skin ageing process is caused by natural ageing, as an intrinsic factor which causes changes in skin elasticity over time, but also by extrinsic factors as the exposure to solar radiation and contamination^7–9^. Both these factors have an impact on collagen and elastin, main responsible for tensile strength and elasticity of the skin, which are denaturated causing the loss of skin integrity^7–9^.

Cumulative UV radiation causes degeneration in the 3D structure of elastic fibers, resulting in the loss of skin elasticity and eventually wrinkle formation, especially in those areas where skin is thinner, like the forehead or eye corners. Also, age diminishes skin thickness, so loss of elasticity is more pronounced over the years^10^. This process is greatly associated with the fibroblast elastase, a member of the chymotrypsin family of proteases which is responsible for the breakdown of elastin and collagen. Under normal conditions, elastase activity is indispensable to enable tissue repair after wounding, but its upper expression due to intrinsic or extrinsic factors increases loss of elasticity and consequently skin damage^11^. Nevertheless, there are several molecules and peptides which can interfere with elastase activity, and thus mitigate the tissue damage caused by UV radiation, contamination, and natural ageing. Regarding the valorization of brewery by-products, this article analyzes the potential of α and β-acids and prenylflavonoids, main phenolic constituents of hops, as bioactive molecules which can diminish elastase activity and prevent wrinkle formation and loss of strength.

To increase the searching possibilities of elastase inhibitors, computational-aided methods can be employed, so this technology has made possible to test an almost unlimited number of drug-like compounds in an automatic way, reducing the wet lab assays^12^. Hence, the use of computational-aided drug design software (CADD) for the discovering of bioactive molecules is nowadays a common approach for the pharmaceutical industry, but it is also useful for cosmetics and even the food and beverages industries. Accordingly, a computational method based on molecular docking and molecular dynamics is employed to examine the elastase receptor against the proposed α and β-acids and prenylflavonoids, detecting those molecules which offer a higher inhibition capacity of elastase and consequently, help to maintain tensile strength and elasticity of the skin.

Once these high-potency anti-elastase molecules were detected, several extracts were prepared with BSH. Different extraction conditions and agents were analyzed, using solvents as water and 100% plant-based propanediol produced through fermentation of corn starch, that accomplish green procedures. Water is the universal solvent, but there are especially polar molecules that are only very sparingly soluble in water and need the help of solvents. Biobased propanediol is a natural, skin-friendly, preservative-boosting alternative to petroleum-based glycols for formulating versatile and innovative cosmetic ingredients.

Then, these BSH extracts were tested to assess their phenolic content, antioxidant activity and elastase inhibitory capacity, proving the excellent characteristic of BSH to be employed as high value-added cosmetic ingredient.

## Results and discussion

To maximize the efficacy of the BSH extracts, it is necessary to identify the hop compounds with the highest activity and also to optimize the parameters influencing the production of these extracts so that the highest elastase inhibition capacity can be achieved. Thus, a three-step methodology was carried out, starting with a computational analysis to test the in-silico inhibition capacity of the proposed hop humulones ((iso-)α-acids), lupulones (β-acids) and prenylflavonoids^13^. Next, BSH extracts were prepared following a procedure rooted on a Box–Behnken factorial design to optimize the extraction agent and conditions, and then testing their anti-elastase capacity^14^. Last, to characterize the composition of these extracts, a HPLC–MS/MS analysis was performed to guarantee the presence of those molecules that were previously identified as potent inhibitors by the computational methodology.

### Molecular modeling: in-silico identification of potent inhibitors

A blind docking analysis was employed to detect the region of interaction of the elastase receptor against the proposed α and β-acids and prenylflavonoids. The elastase binding site is well-known and is stablished by the key residues Hys57, Asp102 and Ser195^15^, so this analysis allows to detect whether those α and β-acids and prenylflavonoids are able to interact with the binding site or whether they remain attached to non-active areas of the receptor.

Accordingly, this study is carried out with the α-acids adhumulone, cohumulone and n-humulone, iso-α-acids cis-iso-α-humulone, cis-iso-α-cohumulone, trans-isocohumulone, trans-isohumulone, iso-α-cohumulone, iso-α-adhumulone, cis-tetrahydroisocohumulone, cis-tetrahydroisohumulone, trans-isocohumulone and trans-tetrahydroisohumulone, trans-tetrahydroisocohumulone, the β-acids lupulone and colupulone and the prenylflavonoids 6-prenylnaringenin, 8-prenylnaringenin, 6-geranylnaringenin, desmethylxanthohumol, xanthohumol and the isomerized form isoxanthohumol. Blind docking performs a conformational analysis of each ligand on all α-carbons of the studied protein. In this way, all possible ligand-receptor binding sites are evaluated, and a score is assigned to quantify the suitability of the binding. In other words, all binding positions and their corresponding binding energy are analyzed, establishing a quantitative ranking of the most favourable positions for each α and β-acid and prenylflavonoid in the elastase receptor^16^.

The interaction of the aforementioned ligands into the elastase binding site is reported in Table 1. The suitability of these positions is identified by a cluster number (CL), being CL1 the most favourable and CLn the most unfavourable (n = 1 – total number of dockings). Favourable bond energies were detected for every ligand inside the inspected binding site, and also the binding site was reached within first three dockings (n CL ≤ 3) so it can be considered that these ligands have a high potential to bind elastase.

**Table 1:** Blind docking analysis of α and β-acids and prenylflavonoids against elastase.

Code	Molecule	CL	E_AD_ (kcal/mol)	E_PV_ key res. (kcal/mol)
117231	Iso-α-adhumulone	1	− 6.12	− 3.5
136636	6-prenylnaringenin	2	− 6.96	> − 1.0
168080	Adhumulone	1	− 6.49	− 3.5
391214	n-Humulone	1	− 6.10	− 3.5
421848	8-prenylnaringenin	3	− 6.69	− 5.5
513197	Isoxanthohumol	2	− 6.59	− 3.5
555077	Xanthohumol	2	− 6.96	–
4653755	6-geranylnaringenin	1	− 7.79	− 3.5
4947362	Desmethylxanthohumol	3	− 6.78	− 3.5
1175902	Trans-isohumulone	2	− 5.96	− 8.0
13433819	Lupulone	1	− 5.65	− 4.0
19994349	Cohumulone	1	− 6.12	− 3.5
20009040	Colupulone	1	− 5.87	− 3.5
21671995	Cis-tetrahydroisocohumulone	1	− 5.90	− 3.5
25015707	Cis-tetrahydroisohumulone	1	− 5.87	− 10.0
29368047	cis-iso-α-humulone	1	− 6.18	− 3.5
32699658	Cis-iso-α-cohumulone	2	− 6.19	− 9.0
57464007	Trans-tetrahydroisohumulone	1	− 5.76	− 10.0
58473849	Trans-isocohumulone	1	− 6.01	− 2.0
58827673	Trans-tetrahydroisocohumulone	2	− 5.59	− 3.5
58575	Epigallocatechin gallate	3	− 7.39	− 5.0
5280343	Quercetin	3	− 6.76	− 5.5

*CL* cluster number, *AD* AutoDock, *PV* PoseView. Employed software: AutoDock, PoseView.

**Figure 1:**
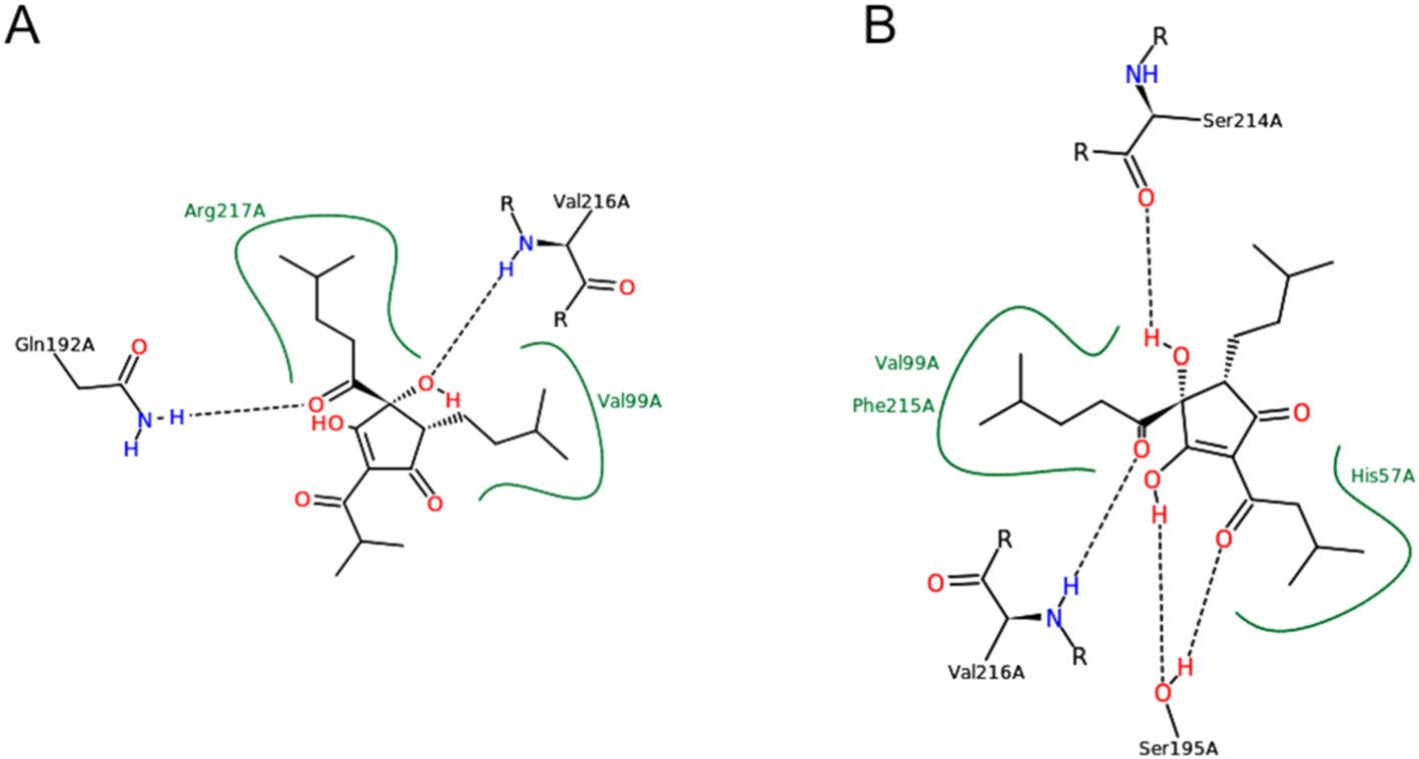
Blind docking of elastase and (**A**) Cis-tetrahydroisocohumulone, (**B**) Cis-tetrahydroisohumulone. (**A**) Shows an incorrect positioning of cis-tetrahydroisocohumulone in the binding site, not being able to interact with the key residues His57, Asp102 nor Ser195, while (**B**) shows the interaction of cis-tetrahydroisohumulone, structurally similar to cis-tetrahydroisocohumulone, bound to the key residues His57 and Ser195.

These molecules have significant structural differences, so there is no common pattern in the way they interact with the receptor and, therefore, they are not always able to interact with the same key residues. Among them, cis- and trans-tetrahydroisohumulone, cis-iso-α-cohumulone and trans-isohumulone have the most favourable key residue binding energies, followed by 8-prenylnaringenin and lupulone. This high affinity seems to be caused by the hydroxy groups, which are correctly positioned to function as acceptor groups and bind the key residues (Figs. 1, Fig. si. 1–22).

The presence of α and β-acids and prenylflavonoids is determined by HPLC–MS/MS in different samples of hop and BSH extracts to determine and quantify those molecules which are present in a more pronounced way (Table 2). Isoxanthohumol, xanthohumol, 8-prenylnaringenin, iso-α-adhumulone and/or n-humulone, iso-α-cohumulone, trans and/or cis-tetrahydroisocohumulone, tetrahydroisohumulone and/or tetrahyroisoadhumulone, lupulone and colupulone were detected as major α and β-acids and prenylflavonoids from hops, being iso-α-adhumulone and/or n-humulone, iso-α-cohumulone and lupulone in higher concentration (Table 2). Several publications state that the most common form, at slightly acidic pH (pH = 5.8–7), is the cis-form^17^. Therefore, it is believed that cis- isomers have higher concentration in those cases where it is not possible to differentiate between these two molecules by mass spectrometry analysis methodologies.

**Table 2:** LC-PDA-LTQ FT Orbitrap mass spectrometry analysis of hop and BSH extract samples.

Sample	Isoxanthohumol	Xanthohumol	8-Prenylnaringenin	Iso-α-ad/n-humulone	Iso-α-cohumulone	Trans/Cis-Tetrahydroiso cohumulone	Tetrahydroiso (ad)humulone	Lupulone	Colupulone
Perle	66.8	96.2	30.1	2018.0	1074.3	0.00	0.00	1142.3	872.1
Nugget	59.5	84.8	28.9	2475.1	1227.8	0.00	0.00	714.7	412.8
BSH 2W	3.22	3.16	< 0.10	4382.0	196.0	3.78	3.20	93.9	59.2
BSH 5W	3.17	2.10	< 0.10	3346.0	181.0	< 0.10	1.52	65.1	36.9
BSH 8W	3.60	3.11	< 0.10	2828.0	193.0	< 0.10	< 0.10	44.4	31.8
BSH 7	10.4	67.4	1.71	2514.0	103.0	< 0.10	< 0.10	62.6	49.4
BSH 7′	13.3	96.9	3.51	2383.0	93.0	< 0.10	< 0.10	115.6	74.6
BSH 12	14.4	90.3	3.52	27,206.0	1186.0	< 0.10	< 0.10	21,924.0	15,619.0

α and β-acids and prenylflavonoids concentration in mg/L.

If the BD results (Table 1) and concentrations of these molecules in hops are considered (Table 2), the most important would be trans-isohumulone, followed by cis-iso-α-cohumulone and lupulone. Thus, a molecular dynamics analysis is carried out with these molecules which offer the best BD results. Also, these other molecules which are in much higher concentration in hop samples were analyzed, because although they may not be the most active, the large difference in concentration may compensate this expected lower bioactivity. Accordingly, trans-isohumulone, cis-iso-α-cohumulone, lupulone, cis- and trans-tetrahydroisohumulone, and 8-prenylnaringenin were selected to test their performance in a dynamic environment, taking into consideration the bound protein residues and their stability within the binding site.

MD results show that trans-isohumulone (Fig. s.i.25) has a low RMSD value for elastase and higher but constant for ligand. This molecule interacts with the key residues Hys57 and Ser195, being the first one an intermittent and unstable contact. Ser195 and the non-key residues Arg61 and Gly193 are the most durable contacts over time, with an intermittent interaction from 10 to 50 ns.

**Figure 2:**
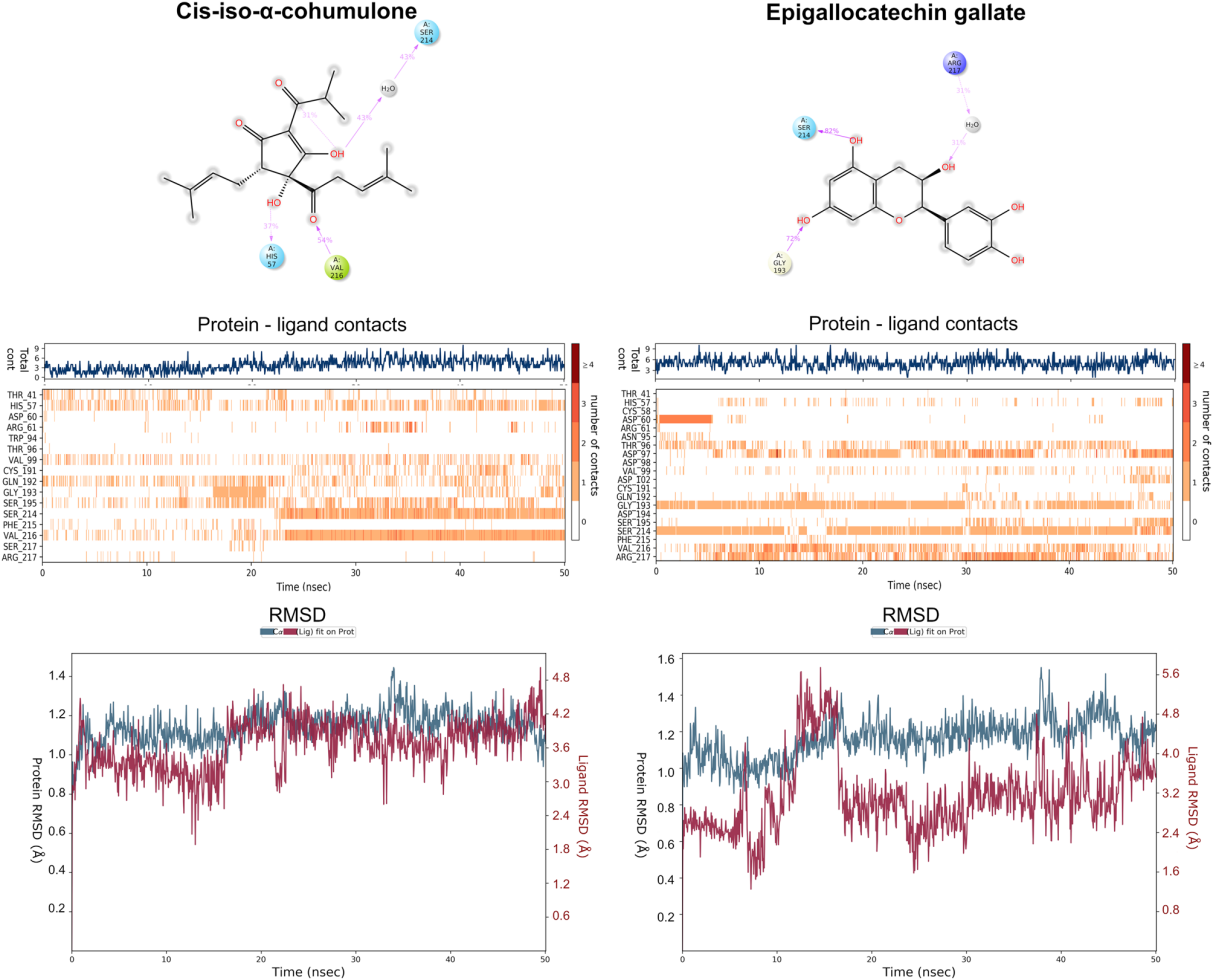
Molecular dynamics analysis of cis-iso-α-cohumulone and the standard elastase inhibitor epigallocatechin gallate. Final docking position, protein–ligand contacts, and RMSD values are represented.

Cis-iso-α-cohumulone (Fig. 2) shows low and constant RMSD values both for protein and ligand, especially from 15 ns onwards. It interacts slightly intermittently, but during the whole simulation period, with the key residues Hys57 and Ser195. Besides, the non-key contacts Val99, Gln92, Ser214 and Val216, which are maintained during the whole simulation help to fix the ligand in a high stable position. Ser214 and Val216 play a major role, so they are permanent contacts after the first 25 ns, and this is a key aspect to promote the interaction with the aforementioned key residues.

Lupulone (Fig. s.i.27) has high and unstable RMSD values for ligand, especially at the end of the simulation period. Regarding the evolution, it can be assumed that this ligand turns from one position to another during the course of the simulation. No interaction was detected with the key residues, and, as it was assumed due to the high RMSD instability, the protein–ligand contacts show that there is an important conformational change of the ligand, which turns from a first interaction with Asn25, Tyr117 and Gln119 to an interaction with Trp27, Tyr137, Gln157 and Tyr207.

Trans-tetrahydroisohumulone (Fig. s.i.28) shows low and constant RMSD values for both protein and ligand throughout the simulation, especially from 8 ns onwards. It only interacts very intermittently and during the first 20 ns of simulation with the key residue His57, although this interaction is practically negligible. Regarding non-key residues, Val216, stable throughout the simulation, and Arg217, slightly more intermittent, stand out. It also shows more intermittent interactions, but throughout the simulation, with Val99, Gln192 and Phe215.

Cis-tetrahydroisohumulone (Fig. s.i.29) has low and constant RMSD for protein throughout the entire simulation. The ligand has a slightly elevated RMSD from 15 ns onwards, at which point a conformational change that modifies its binding to the receptor occurs. This ligand interacts highly intermittently and only in the first 20 ns of the simulation with the key residues His57 and Ser195, although these interactions are practically negligible. All ligand interactions are rather intermittent, except with the non-key residues Ala99, Trp172 and Phe215, whose interaction remains constant especially from 20 ns onwards.

8-prenylnaringenin (Fig. s.i.30) has low RMSD for both protein and ligand, especially from 20 ns onwards. It interacts intermittently throughout the simulation with the key residue His57 and more constantly with the key residue Ser195. It also has slightly intermittent interactions during the whole simulation with the non-key residues Gly193, Ser214, Val216 and Arg217, giving the ligand a high stability.

Two standard references as quercetin and epigallocatechin gallate (EGCG) were tested to contrast their performance against these molecules. Quercetin (Fig. s.i.26) shows slightly high RMSD values around 6.5 Å, and interacts intermittently but during the whole MD simulation with His57. It also interacts with Ser195, but only in certain moments. As non-key residues, quercetin interacts permanently with Val216 and Arg217, and intermittently but throughout the entire simulation with Thr96, Val99, Thr175 and Phe215. This high number of non-key contacts helps to stabilize the ligand and makes quercetin a potent elastase inhibitor.

Epigallocatechin gallate (Fig. 2) shows a really low RMSD value around 3.5 Å and interacts with the three key residues His57, Asp102 and Ser195. The most relevant contact is His57, which is intermittent but maintained during the whole simulation. Asp102 is maintained during the last 5 ns and Ser195 is also intermittent but maintained throughout the entire simulation, especially during the last 30 ns. As non-key residues, epigallocatechin gallate interacts permanently with Gly193 and Ser214, and also slightly more intermittently with Thr96, Asp97, Val216 and Arg217, making this molecule a high stable ligand and thus a strong elastase inhibitor.

According to these MD results, cis-iso-α-cohumulone and 8-prenylnaringenin are the most stable ligands (Figs. 2, Fig. s.i.30). Compared against the standard inhibitors, these two molecules show a similar MD behaviour to quercetin and epigallocatechin gallate (Figs. 2, Fig. s.i.26), so they are expected to act as elastase inhibitors. Cis-iso-α-cohumulone, which was detected as the most potent inhibitor by MD analysis (Fig. 2), is also present in high concentration in hop samples (Table 2), so probably this molecule would inhibit elastase in a more effective way than 8-prenylnaringenin due to this higher concentration.

### Optimization and characterization of BSH extracts

**Figure 3:**
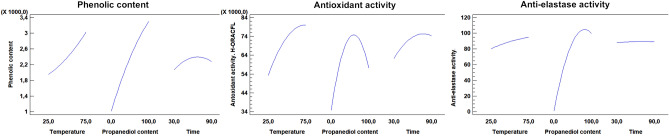
Phenolic content, antioxidant activity and elastase inhibition percentage evolution related to propanediol content, extraction time and extraction temperature.

To contrast these theoretical results, BSH extracts were prepared using propanediol-water mixtures (Table s.i.2) following a Box–Behnken factorial design (Fig. s.i.23) combined with the response surface methodology (RSM) which is explained on “Preparation of hop extracts” section^14^. Also, an experimental design using water as extraction solvent was performed (Table s.i.2). Results show that temperature and propanediol percentage improve the phenolic content, antioxidant activity and elastase inhibition (Fig. 3, Table s.i.1). Water extraction procedures, despite of being able to achieve high concentrated phenolic extracts with good antioxidant capacities, do not show anti-elastase activity, regardless of time or temperature used during the extraction process (Table s.i.2). In this case, epigallocatechin gallate was taken as a control standard. Between the two standard inhibitors computationally tested, quercetin and epigallocatechin gallate, the latter was chosen because it has a molecular structure of similar complexity to the bioactive compounds present in hops.

The desirability function of the RSM was used to optimize the extraction variables on the process of obtaining bioactive extracts, seeking to maximize multiple responses (phenolic content and/or antioxidant activity and/or anti-elastase activity) (Fig. s.i.24). The optimal operating factors to maximize antioxidant capacity and anti-elastase activity were achieved at 74 °C, 79.92% propanediol and 51.45 min extraction. BSH extract 18 obtained under these conditions shows 100% elastase inhibition capacity, antioxidant capacity equivalent to 81.9 mmol Trolox/L and a high phenolic content (Table 3). These predicted values are close those obtained for BSH extract 14 under conditions that also maximize the phenolic content (Table 3).

**Table 3:** Optimization procedure using RSM to maximize phenolic content and/or antioxidant activity and/or anti-elastase activity.

BSH extract	Optimal conditions of RSM model			Predicted response values		
	Temperature (°C)	Propanediol content (%)	Time (min)	Phenolic content (mg GAE/L)	Antoxidant activity H-ORAC_FL_ (mmol TE/L)	Anti-elastase activity (% inhibition)^a^
14	75	98.75	77.91	**4700**	**78.07**	**100.0**
15	75	100.0	60.60	4660	75.58	**100.0**
16	74	79.92	51.45	3940	**81.90**	100.0
17	75	98.64	77.87	**4696**	**78.13**	100.0
18	74	79.92	51.45	3940	**81.90**	**100.0**

Bold values shows optimized variable(s) for each extract.

^a^The effective or inhibitory concentration that reduces the enzyme activity to 50% (EC_50_ value) of the control EGCG is 2.3 mg/L (experimental data, n = 3).

Epigallocatechin gallate was taken as a control standard for anti-elastase activity assay, being the effective or inhibitory concentration that reduces the enzyme activity to 50% of the control EGCG (EC_50_ value) 2.3 mg/L (experimental data, n = 3). On the other hand, BSH 14 reduces de activity of elastase to 100% at 4.7 mg GAE/L total phenolic concentration (predicted data). Between the two molecules computationally tested, quercetin and epigallocatechin gallate, the latter was chosen because it has a molecular structure of similar complexity to the bioactive compounds present in hops.

Then, to obtain a quantitative molecular characterization of BSH extracts a HPLC–MS/MS analysis was carried out following the 4.4.2 procedure. These results (Table 2) show that those propanediol-water extracts where cis-iso-α-cohumulone and 8-prenylnaringenin molecules are in higher concentration (BSH 7, 7′ and 12) have higher anti-elastase activity, meanwhile water extracts where there is lower concentration of cis-iso-α-cohumulone and no presence of 8-prenylnaringenin do not have anti-elastase potential (BSH 2W, 5W and 8W). This proves the strong inhibition capacity of these two molecules, which was also previously reported during the computational analysis procedure.

## Conclusions

Following a Box–Behnken factorial design combined with the response surface methodology (RSM), an extract that optimizes elastase inhibition and antioxidant capacity was designed.

Following this process, cis-iso-α-cohumulone and 8-prenylnaringenin were detected as the most potent elastase inhibitors. During the HPLC–MS/MS analysis, both molecules were proved to be present in higher concentration in those samples with major anti-elastase activity (BSH 7, 7′ and 12) and the computational analysis showed a similar behaviour of these molecules compared to the standard inhibitors quercetin and epigallocatechin gallate. Due to the high concentration of cis-iso-α-cohumulone, is expected to be the main responsible for the elastase inhibition on brewers’ spent hop extracts, in comparison to other molecules commonly present in hops is higher concentration as iso-α-adhumulone, n-humolone, lupulone or colupulone.

Thus, it has become clear that the presence of the potent bioactive molecules cis-iso-α-cohumulone and 8-prenylnaringenin gives great potential of BSH to produce high value-added cosmetic products.

## Materials and methods

### Resources and programs

Research Collaboratory for Structural Bioinformatics Protein Data Base (RCSB PDB), UniProt Knowledgebase (UniProtKB), Swiss Protein Database (SwissProt) and Chemspider Database.

Openbabel GUI 2.4.1^18^, Acpype^19^, Gromacs 2018^20–22^, AutoDock Tools 4.2^23^, AutoDock Vina 2.0^24^, Pymol 2.3 (The PyMOL Molecular Graphics System, Version 2.3 Schrödinger, LLC), Python 2.7.6 (Python Software Foundation), PoseView 1.1.2 (ZBH University of Hamburg, BioSolveIT GmbH), Omega 2.5.1.4 (OpenEye Scientific Software)^25^, PLIP 1.3.2^26^, Maestro suite 2020.4, Schrödinger LLC, and the Shuttlemol suite of HPC scripts for Virtual Screening (http://bio-hpc.eu), Statgraphics 19 (Statgraphics Technologies, Inc).

### Materials and reagents

H_2_O 0.1% H-COOH hypergrade for LC–MS LiChrosolv and ACN 0.1% H-COOH hypergrade for LC–MS LiChrosolv were purchased from Sigma Aldrich, as Folin & Ciocalteu’s phenol reagent and Fluorescenin sodium salt Bioreagent, suitable for fluorescence. ZEMEA^®^ Propanediol was purchased from DuPont Tate & Lyle Bio Products Company. Gallic acid ≥ 98% purity, 2,2′-Azobis(2-methylpropionamidine) dihydrochloride (AAPH) ≥ 98% purity and Trolox ≥ 97% purity were purchased from Acros organics. Native human elastase (neutrophil) was purchased from Bio-RAD. Methoxysuccinyl-Ala-Ala-Pro-Val-p-nitroanilide was purchased from Calbiochem, and epigallocatechin gallate, ≥ 98% from Santa Cruz Biotechnology, Inc.

Perle and Nugget hop pellets, brewers’ spent hop and beer samples were supplied by Hijos de Rivera S.A.U. To prepare the hop extracts, a heating plate, thermometer, and glass vessels were employed.

### Molecular modelling

#### Molecular files preparation

The elastase receptor 1qnj was downloaded from RCSB^15^ and ligands were removed from the protein file employing Pymol 2.3. Then, AutoDock Tools 4.2 was used to remove water molecules, add hydrogens, assign AD4 type to all atoms, compute Gasteiger charges and save protein files both in pdb and pdbqt formats^16^.

The α and β-acid and prenylflavonoid molecules 6-prenynlaringenin, adhumulone, cohumulone, cis-isohumulone, cis-isocohumulone, R-humulone, 8-prenylnaringenin, 6-geranylnaringenin, desmethylxanthohumol, xanthohumol, trans-isocohumulone, trans-isohumulone, isocohumulone, isoadhumulone, lupulone, cis-tetrahydroisocohumulone, cis-tetrahydroisohumulone, isoxanthohumol, trans-tetrahydroisohumulone, trans-isocohumulone, trans-tetrahydroisocohumulone, and colupulone were downloaded from Chemspider (http://www.chemspider.com) or Pubchem (http://www.pubchem.ncbi.nlm.nih.gov) in mol format and were converted to mol2, pdbqt and pdb using Pymol 2.3, AutoDock Tools 4.2 and Openbabel GUI 2.4.1.

#### Blind docking analysis

The regions of interaction between the elastase receptor and α and β-acids and prenylflavonoids were detected employing a blind docking study (BD)^27^.

Series of single docking simulations were performed in all protein α-carbon, detecting the most favourable binding sites in terms of affinity between ligand and protein residues but also the spatial conformation of adopted by the ligand into the binding site. AutoDock 4.2 quantifies the affinity of ligand–protein interactions and PoseView 1.1.2 calculates individual energies of each bonded residue, representing global bond energy and key residues bond energy, respectively^16^.

#### Molecular dynamics simulation

The approximation of the behaviour of the system against real conditions is studied by means of a molecular dynamics (MD) analysis. This allows to test the stability of the ligand–protein contacts, previously detected with a BD analysis, during a period of time.

This MD analysis was performed using the GPU version of Desmond included with Maestro suite 2020.4 (Schrödinger LLC) on a workstation with a NVIDIA QUADRO 5000. The system conformed by the ligand and protein of interest was solvated in an aqueous environment, in a cubic box with a minimal distance of 10 Å between the biomolecule and the box boundary (for periodic boundary conditions). Next, systems were neutralized and maintained in 0.15 M NaCl. The OPLS3 force-field and the TIP3P-TIP4P water model were employed. Initially, the systems were simply energy-minimized for 1000-time steps. Then, systems were allowed to execute free dynamics in the NPT ensemble; pressure was controlled using the Martyna-Tobias-Klein methodology and the Nose–Hoover thermostat was employed to maintain the system near 310 K^28^. Production-grade MD trajectories were extended to a total duration of 50-ns per system.

MD trajectories were characterized in terms of the root-mean-square deviation (RMSD) of fluctuations of ligand and receptor, particularly in terms of the main interactions with the top interacting residues. The trajectories were also used to assess the stabilities of the protein secondary structures (in complex with potential inhibitor) by plotting RMSDs^16^.

### Analytical methods

#### Preparation of hop extracts

Hops were contacted with water, propanediol and mixtures of water:propanediol using a solid:liquid ratio of 1:15 (w:v) at several temperatures and contact times. After cooling, the liquid phase was recorded by filtration. A modified Box–Behnken factorial design^14^, 3 factor 3 levels, was used to investigate the effects of three independent variables on the extraction process. The design consisted of 16 experimental points and three replications at the centre point. The independent variables and their considered ranges were propanediol content (0–100%), temperature (25–75 °C) and time (30–90 min). The dependent variables selected in this study were total phenolic content, antioxidant activity (ORAC_FL_) and anti-elastase activity.

Box–Behnken design combined with the response surface methodology (RSM) was used to determine the optimal factors and the targeted response parameters. The experimental data were statistically analyzed by applying commercial software Statgraphics 19 (Statgraphics Technologies, Inc).

#### High pressure liquid chromatography–mass spectrometry–mass spectrometry analysis (HPLC–MS/MS)

For the analysis of hop extracts, a liquid chromatography equipment with an LC1290 Infinity II system coupled to Agilent’s 6546 LC/Q-TOF (Quadrupole-Time-of-Flight) mass spectrometer was used. Agilent Mass Hunter Workstation was employed, being Agilent Mass Hunter Qualitative Analysis 10.0 used for qualitative analysis and Mass Hunter Quantitative Analysis 10.1 for the quantitative analysis of the samples.

The chromatographic conditions were a flow rate of 250 μL/min, injection volume 10 μL, column Kinetex XB-C18 100 × 2.1 mm 2.6 μm Phenomenex at 30 °C temperature, and as mobile phase A: H_2_O 0.1% H-COOH and B: ACN 0.1% H-COOH. The employed gradient was as follows: starting with 98% A and 2% B, in 2 min was changed to 92% A and 8% B. Then, at 12 min it was modified to 80% A and 20% B. In one minute, it was changed to 70% A and 30% B and in another minute, it was changed to 0% A and 100% B. For the three following minutes these conditions were maintained, changing in one minute to the initial conditions, which were maintained for another 7 min.

For the mass conditions a negative ESI was used with a mass range between 50 and 500, the gas temperature was 210 °C and the sheath gas temperature was 350 °C, the sheath gas flow was 11L/min, the drying gas was 13 L/min, and the nebulizer pressure was 35psi. The VCap value was set at 4000 V and the fragmentor at 130 V.

#### Total phenolic content

The total phenolic content was determined according to the Folin-Ciocalteu method based on a procedure described by Medina-Remón et al.^29^. Total polyphenols were expressed as mg Gallic Acid Equivalent (GAE) per litre of sample (mg GAE/L).

#### Antioxidant activity by oxygen radical absorbance capacity (ORAC fluorescein) assay

The original method of Ou et al.^30^ was modified as follows by Dávalos et al.^31^. The reaction was carried out in 75 mM phosphate buffer (pH 7.4), and the final reaction mixture was 200 µL. The mixture of antioxidant and fluorescein was preincubated for 15 min at 37 °C. AAPH (2,20-Azobis (2-methylpropionamidine) dihydrochloride) solution was added. The plate was placed in the reader and the fluorescence recorded every minute for 120 min (excitation wavelength 485 nm, emission wavelength 520 nm). The plate was automatically agitated prior each reading. A blank using phosphate buffer instead of the antioxidant solution and eight calibration solutions using Trolox as antioxidant were also carried out in each assay. Results were calculated on the basis of the differences in areas under the fluorescein decay curve between the blank and the sample, and were expressed as mmol of Trolox Equivalents/L extract (mmol TE/L).

#### Elastase inhibitory assay

The elastase inhibitory activities of extracts were determined according to the method of Azmi et al.^7^ with some modifications via human neutrophil elastase (HNE) hydrolysis of a chromogenic substrate, N-methoxysuccinyl-Ala-Ala-Pro-Val-p-nitroanilide (MAAPVN) at 25 °C in a 96-well plate format. A 25 μL elastase, 100 μL of 0.1 M Tris–HCl (pH 7.5) with 0.5 M sodium chloride and 50 μL of sample were mixed and incubated for 10 min at 25 °C. Then, 25 μL of substrate solution was added and incubated for 60 min at 25 °C^7^. The reaction was monitored by recording the absorbance at 410 nm every minute and inhibition rate was calculated as follows:

$${\text{Inhibition rate }}\left( \% \right) \, = [{1} - \left( {{\text{C}} - {\text{D}}} \right)/\left( {{\text{A}} - {\text{B}}} \right)]\cdot{1}00$$

where A indicates the absorbance at 410 nm without a test sample after incubation, B indicates the absorbance at 410 nm without a test sample before incubation, C indicates the absorbance at 410 nm with a test sample after incubation, and D indicates the absorbance at 410 nm with a test sample before incubation. Epigallocatechin gallate was used as a positive control.

## Supplementary Information

Supplementary Information.

## Data Availability

All data generated or analyzed during this study are included in this published article and its supplementary information.
