# Supplementary material for: Brewers’ spent hop revalorization for the production of high added-value cosmetics ingredients with elastase inhibition capacity
Source: Sci Rep. 2022 Dec 21;12:22074. doi: 10.1038/s41598-022-26149-3 (PMC9772169; doi:10.1038/s41598-022-26149-3)
Supplement: Supplementary file 1 — Supplementary Information. [file 41598_2022_26149_MOESM1_ESM.docx]

**Brewers’ spent hop revalorization for the production of high added-value cosmetics ingredients with elastase inhibition capacity**

Paredes-RamosMaria^a*^, Conde PiñeiroEnma^b^, Lopez VilariñoJose M.^a^

^a^Hijos de Rivera S.A.U., C/ José María Rivera Corral 6, A Coruña, Spain

^b^GLECEX S.L. (Global and Ecofriendly Natural Extracts S.L.), Edificio CITI, Parque Tecnolóxico de Galicia, San Cibrao das Viñas, Ourense, Spain

*corresponding author: [mparedes@estrellagalicia.es](mailto:mparedes@estrellagalicia.es)


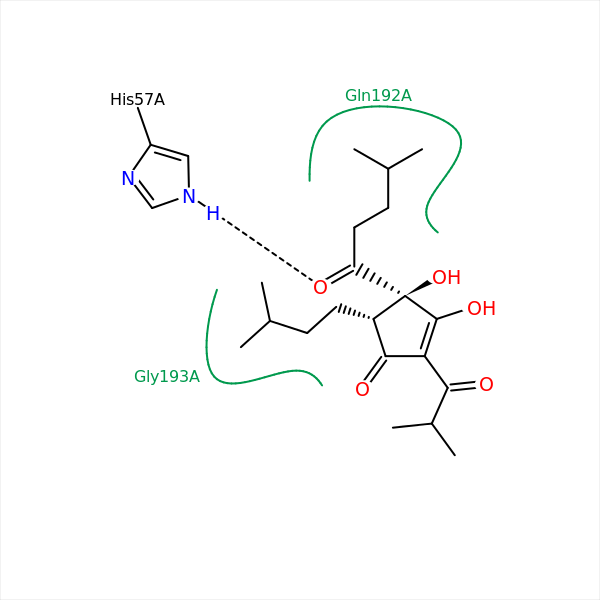


Figure s.i. 1. Trans-tetrahydroisocohumulone


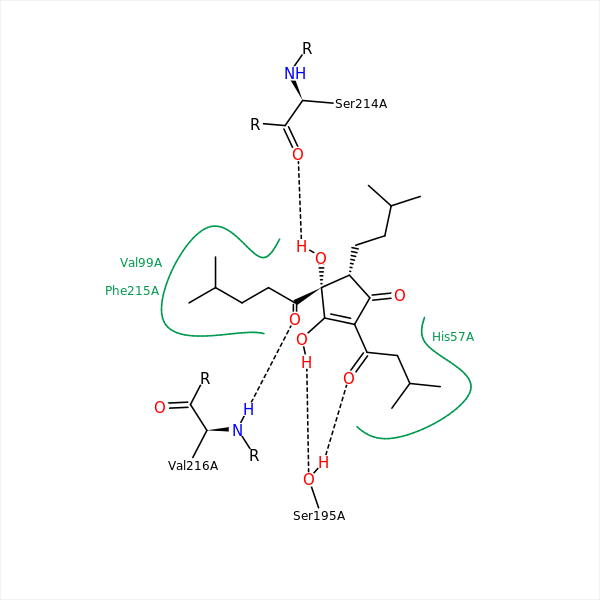


Figure s.i. 2. Cis-tetrahydroisohumulone


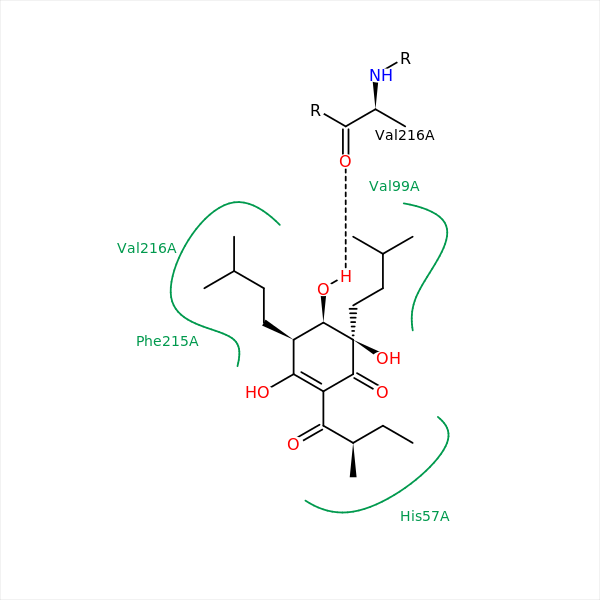


Figure s.i. 3. Adhumulone.


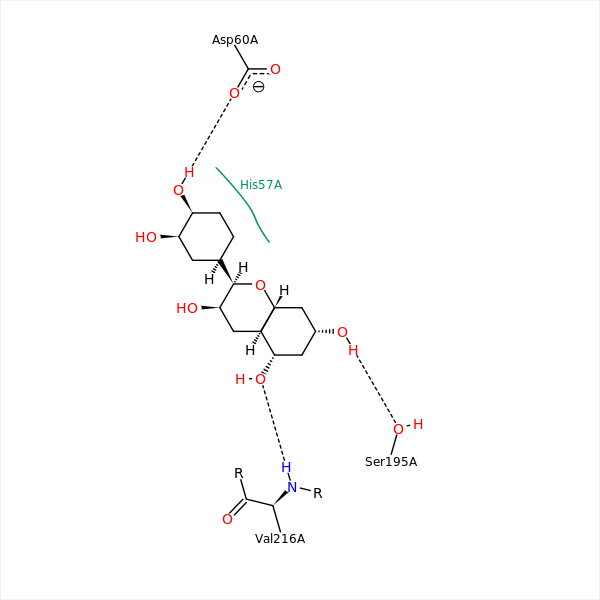


Figure s.i. 4. Epigallocatechin gallate


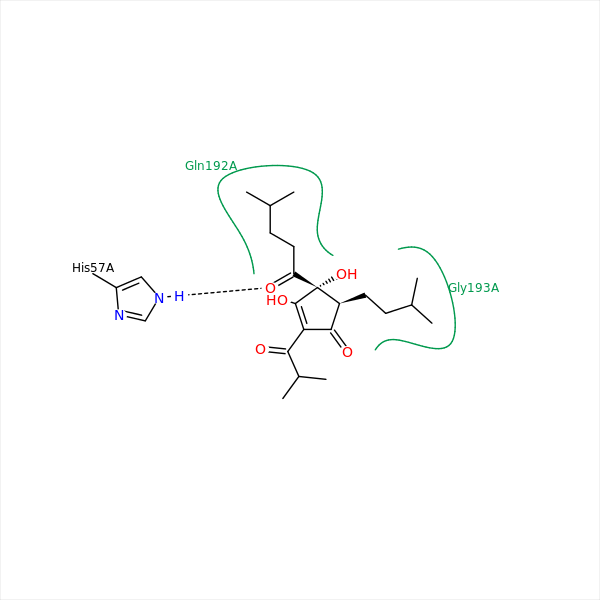


Figure s.i. 5. Trans-isocohumulone


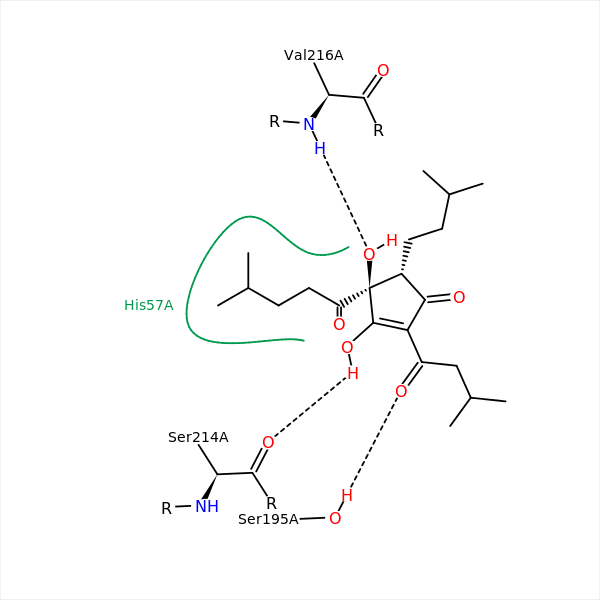


Figure s.i. 6. Isoadhumulone


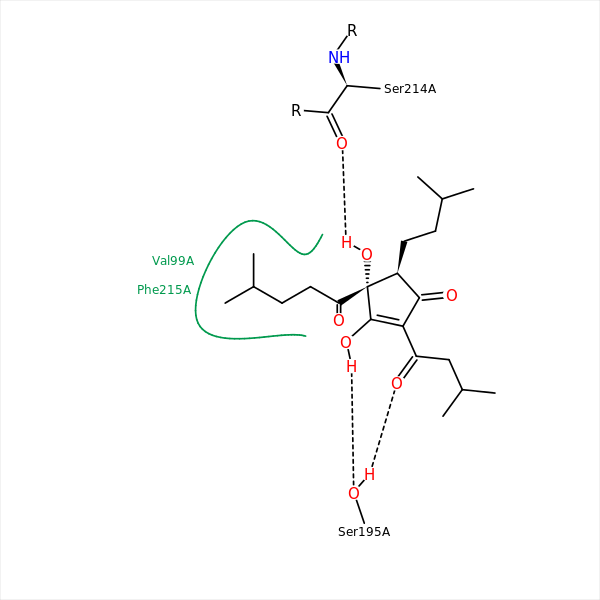


Figure s.i. 7. Trans-tetrahydroisohumulone


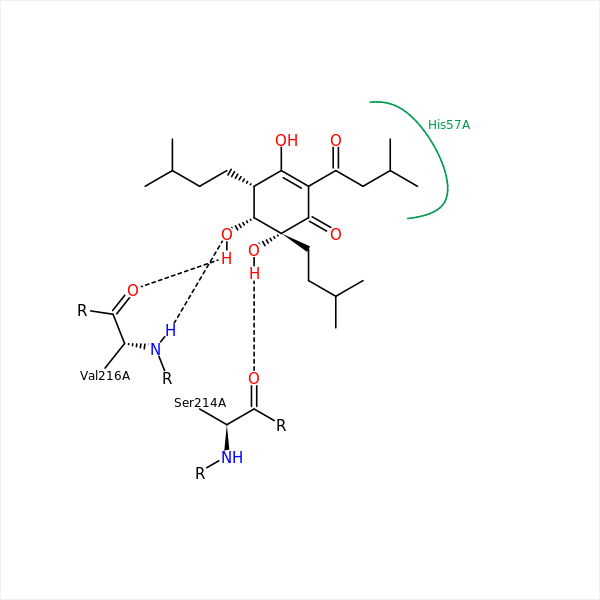


Figure s.i. 8. R-Humulone


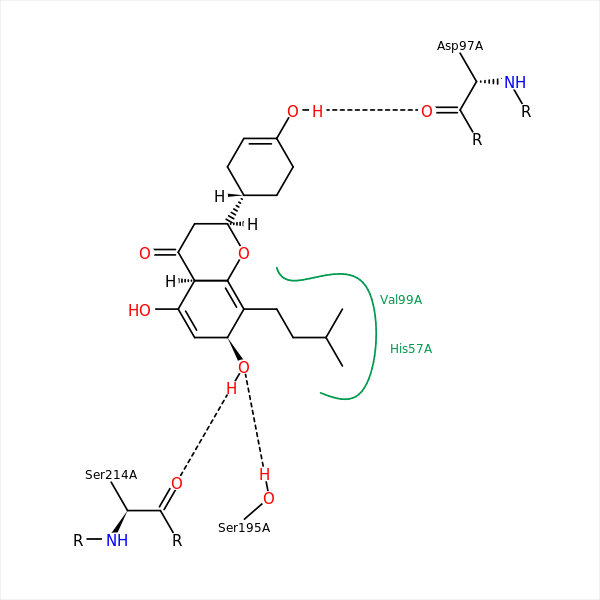


Figure s.i. 9. 8-prenylnaringenin


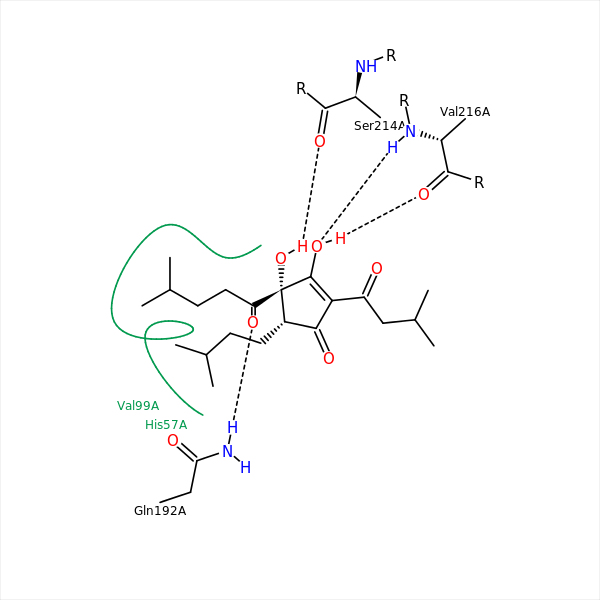


Figure s.i. 10. Cis-isohumulone


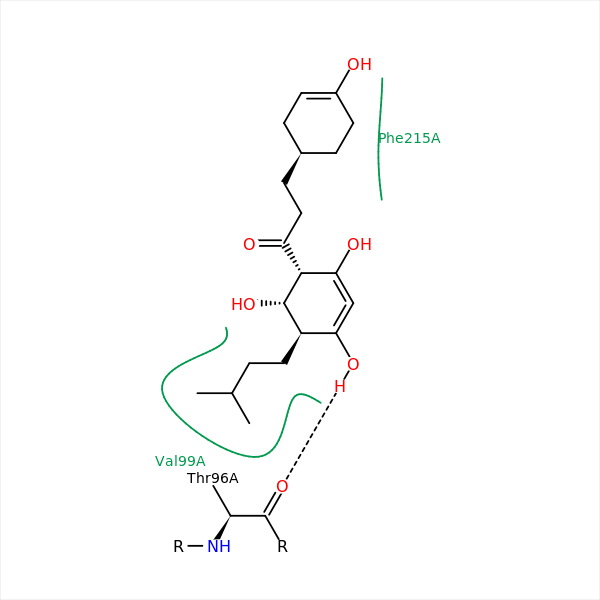


Figure s.i. 11. Desmethylxanthohumol


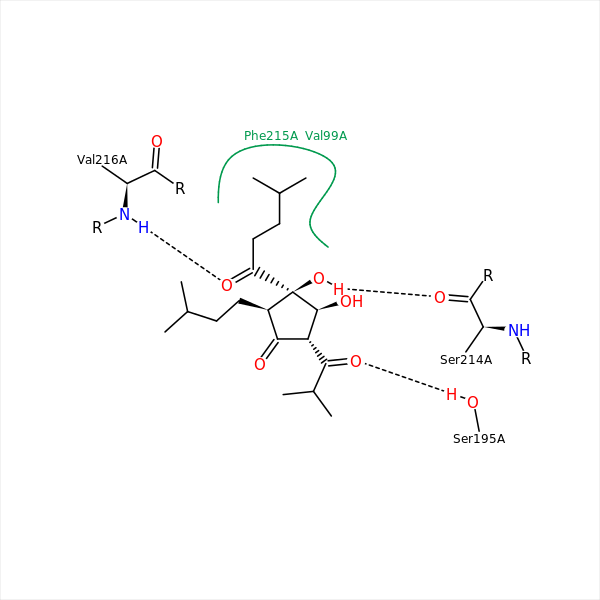


Figure s.i. 12. Cis-isocohumulone


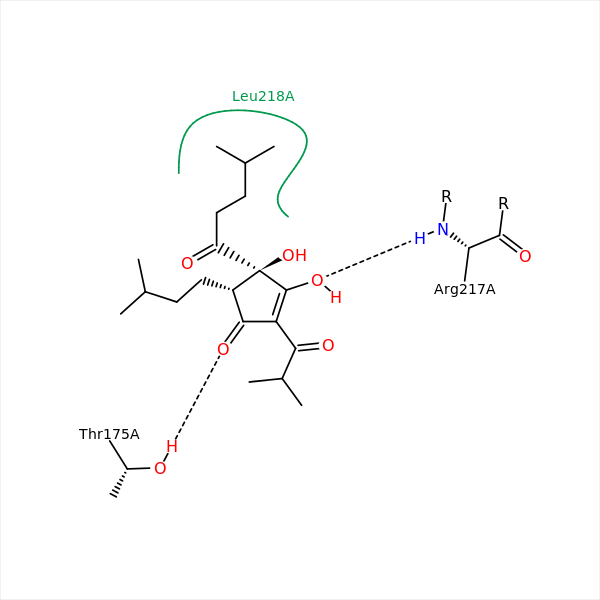


Figure s.i. 13. Trans-tetrahydroisocohumulone


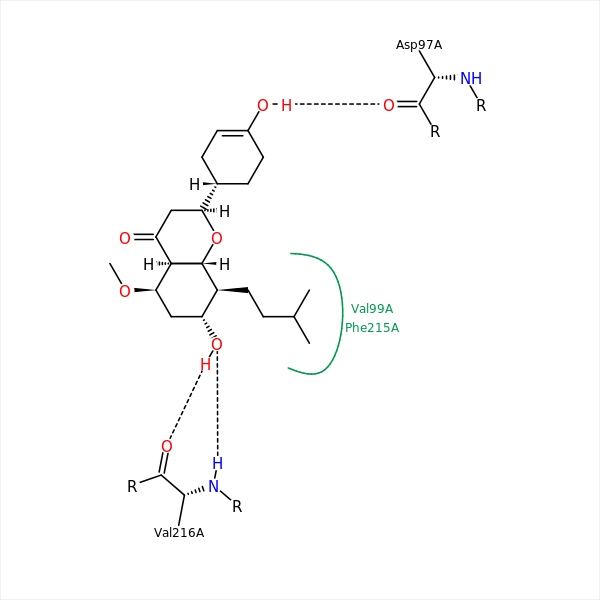


Figure s.i. 14. Isoxanthohumol


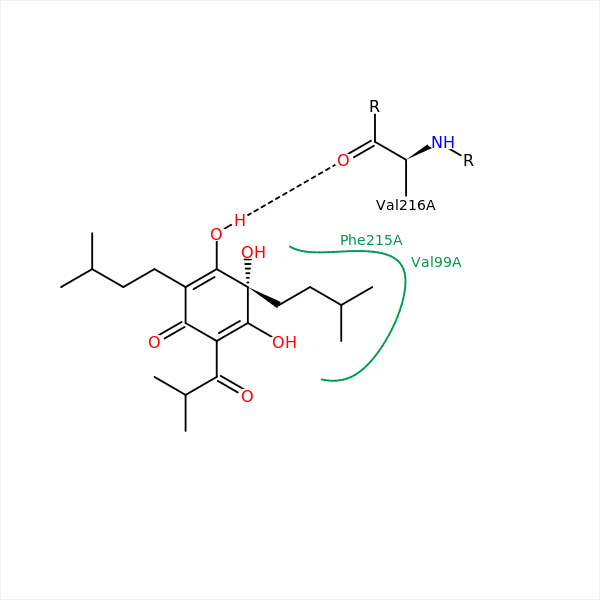


Figure s.i. 15. Cohumulone


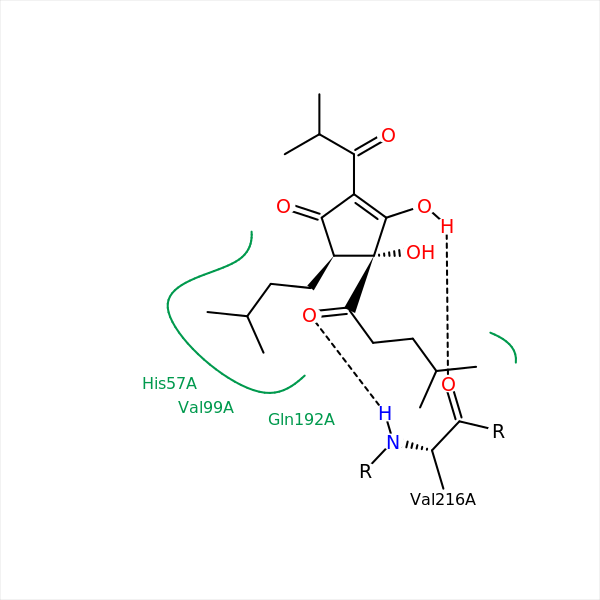


Figure s.i. 16. Trans-tetrahydroisocohumulone


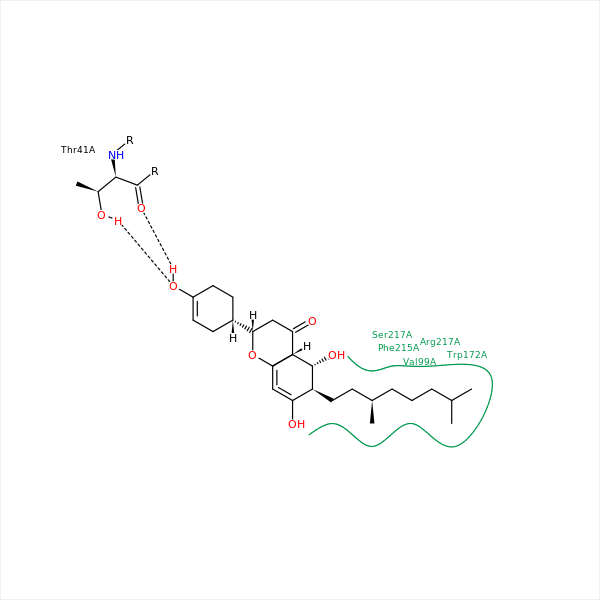


Figure s.i. 17. 6-geranylnaringenin


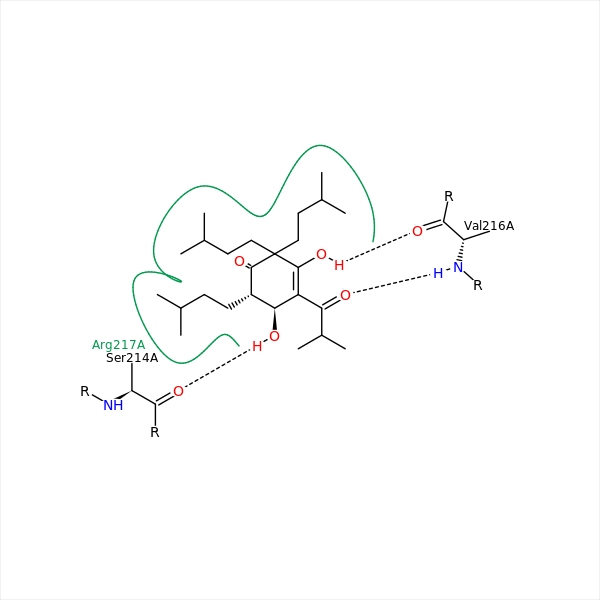


Figure s.i. 18. Colupulone


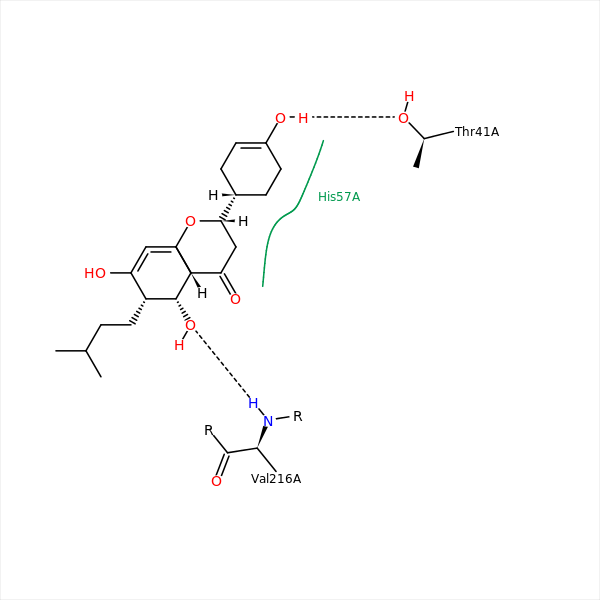


Figure s.i. 19. 6-prenylnaringenin


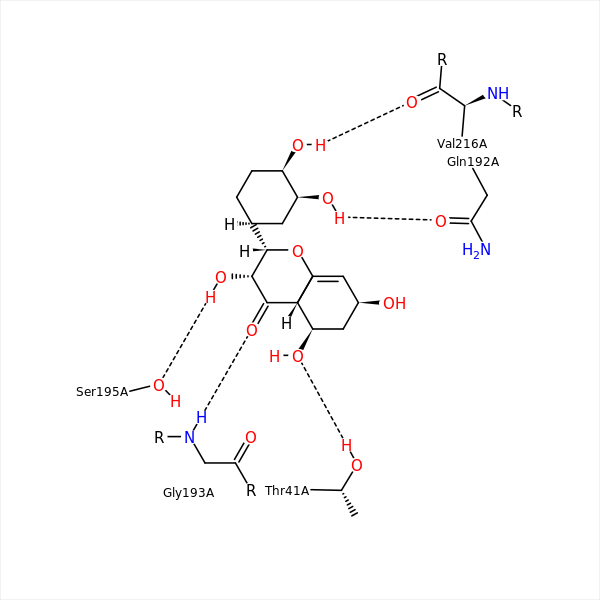


Figure s.i. 20. Quercetine


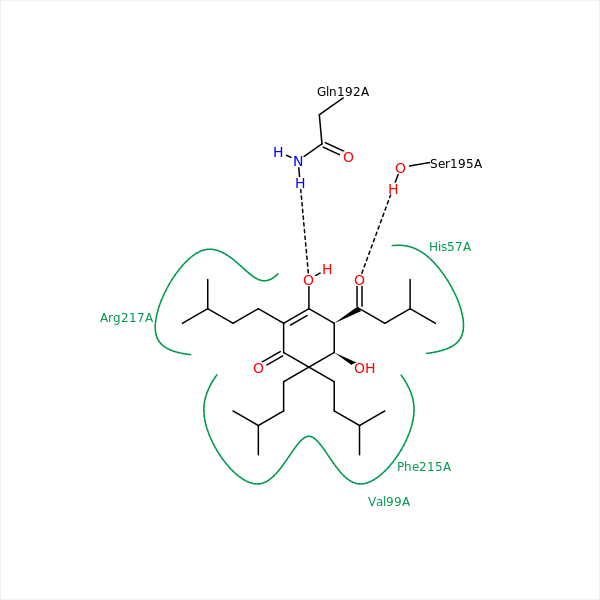


Figure s.i. 21. Lupulone


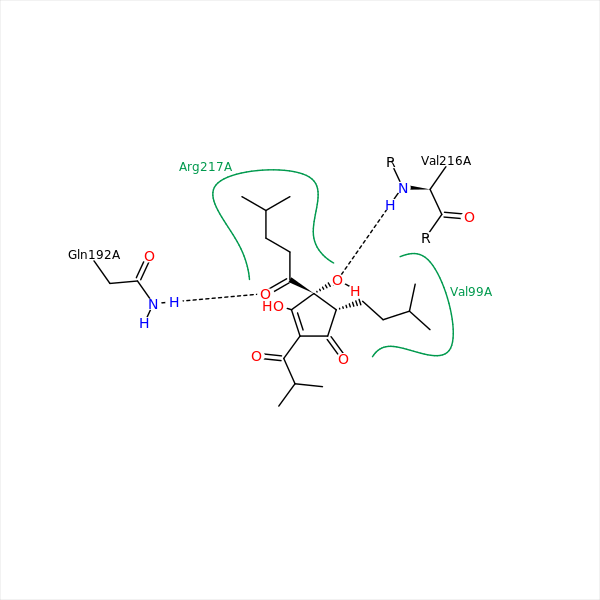


Figure s.i. 22. Cis-tetrahydroisocohumulone

Figure s.i. 23. Box-Behnken design for brewers’ spent hop extracts

Table s.i. 1. Box-Behnken design for brewers’ spent hop extracts prepared with propanediol-water mixtures.

|  | **INDEPENDENTVARIABLES** | | | **I.V. CODE** | | | **RESULTS** | | |
| --- | --- | --- | --- | --- | --- | --- | --- | --- | --- |
| **Design points** | **T (̊C)** | **Propanediol content (%)** | **Time (min)** | **T** | **Propanediol content** | **Time** | **Phenolic content**  **(mg GAE/L)** | **Antioxidant activity H-ORAC_FL_**  **(mmol Trolox/L)** | **Anti-elastase activity**  **(% inhibition)** |
| 1 | 25 | 50 | 30 | -1 | 0 | -1 | 1,979.1 | 44,25 | 72.2 |
| 2 | 25 | 0 | 60 | -1 | -1 | 0 | 854.7 | 21.68 | 0.0 |
| 3 | 25 | 100 | 60 | -1 | 1 | 0 | 2,097.6 | 22.39 | 93.8 |
| 4 | 25 | 50 | 90 | -1 | 0 | 1 | 2,005.4 | 58.88 | 75.3 |
| 5 | 50 | 0 | *30* | 0 | -1 | -1 | 954.7 | 26.76 | 0.0 |
| 6 | 50 | 100 | 30 | 0 | 1 | -1 | 2,453.4 | 36.43 | 98.4 |
| 7 | 50 | 50 | 60 | 0 | 0 | 0 | 2,407.2 | 75.63 | 85.2 |
| 7' | 50 | 50 | 60 | 0 | 0 | 0 | 2,467.9 | 67.96 | 94.2 |
| 7'’ | 50 | 50 | 60 | 0 | 0 | 0 | 2,270,0 | 77.50 | 89.4 |
| 8 | 50 | 0 | 90 | 0 | -1 | 1 | 1,011.7 | 34.76 | 0.0 |
| 9 | 50 | 100 | 90 | 0 | 1 | 1 | 3,386.0 | 64.11 | 100.0 |
| 10 | 75 | 50 | 30 | 1 | 0 | -1 | 2,666.9 | 72.43 | 100.0 |
| 11 | 75 | 0 | 60 | 1 | -1 | 0 | 1,049.5 | 31.29 | 0.0 |
| 12 | 75 | 100 | 60 | 1 | 1 | 0 | 5,083.2 | 80.43 | 100.0 |
| 13 | 75 | 50 | 90 | 1 | 0 | 1 | 2,472.3 | 69.42 | 100.0 |


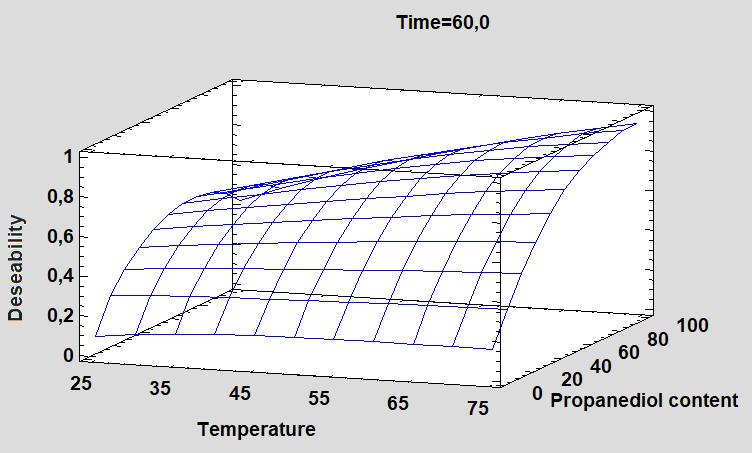


Figure s.i. 24. Desirability in optimization procedure to maximize phenolic content, antioxidant activity and/or anti-elastase activity.

Table s.i. 2. Design for brewers’ spent hop extracts prepared with water.

|  | **INDEPENDENTVARIABLES** | | | **I.V. CODE** | | | **RESULTS** | | |
| --- | --- | --- | --- | --- | --- | --- | --- | --- | --- |
| **Design points** | **Temperature (̊C)** | **Propanediol content (%)** | **Time (min)** | **Temperature (̊C)** | **Propanediol content (%)** | **Time (min)** | **Phenolic content (mg GAE/L)** | **Antioxidant activity. H-ORACFL (mmol Trolox/L)** | **Anti-elastase activity (% of inhibition)** |
| 1W | 25 | 0 | 30 | -1 | - | -1 | 817.8 | 20.81 | 0.0 |
| 2W | 25 | 0 | 60 | -1 | - | 0 | 854.7 | 21.68 | 0.0 |
| 3W | 25 | 0 | 90 | -1 | - | 1 | 855.3 | 23.88 | 0.0 |
| 4W | 50 | 0 | 30 | 0 | - | -1 | 954.7 | 26.76 | 0.0 |
| 5W | 50 | 0 | 60 | 0 | - | 0 | 987.5 | 35.93 | 0.0 |
| 6W | 50 | 0 | 90 | 0 | - | 1 | 1,011.7 | 34.76 | 0.0 |
| 7W | 75 | 0 | 30 | 1 | - | -1 | 1,073.2 | 33.91 | 0.0 |
| 8W | 75 | 0 | 60 | 1 | - | 0 | 1,049.5 | 31.29 | 0.0 |
| 9W | 75 | 0 | 90 | 1 | - | 1 | 1,018.4 | 28.16 | 0.0 |


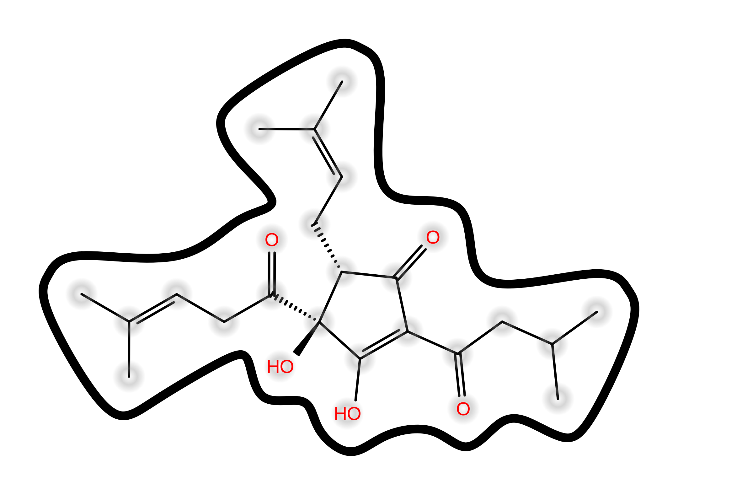


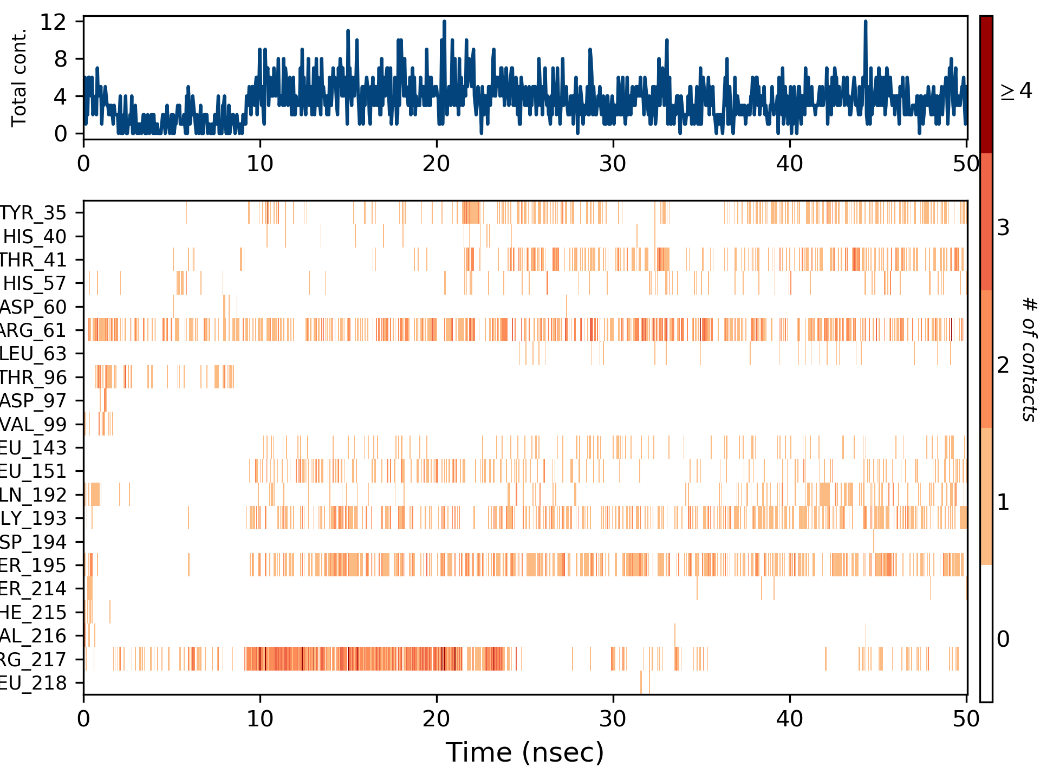


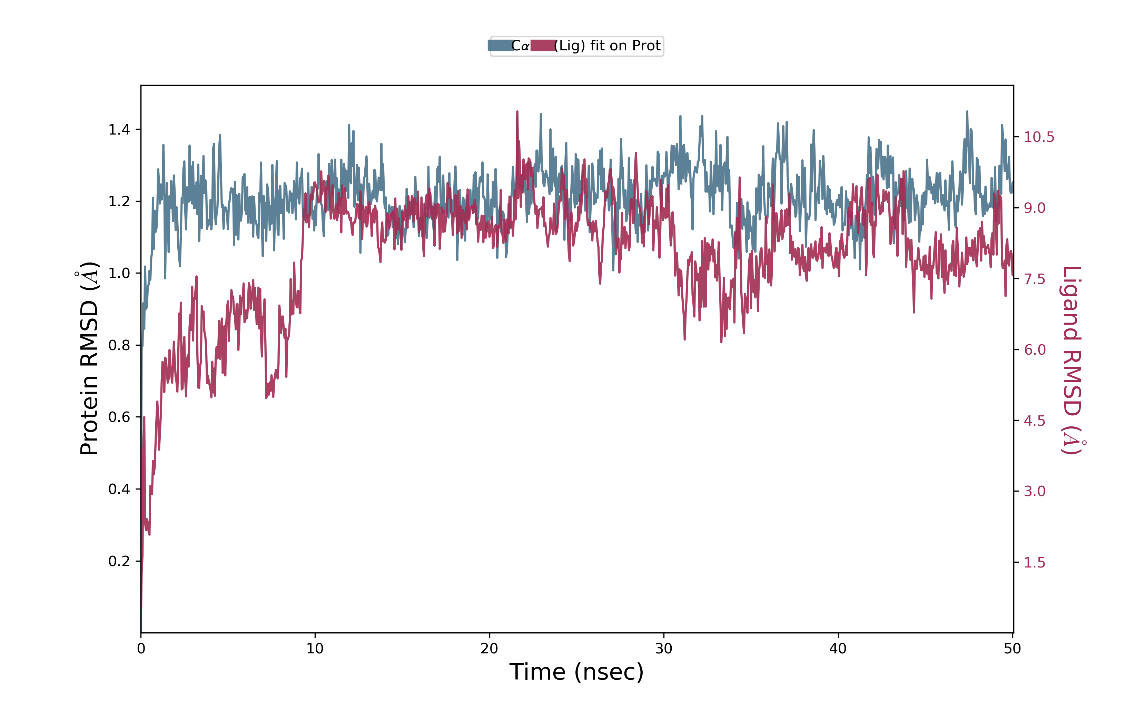


Figure s.i. 25. Molecular dynamics analysis of trans-isohumulone.


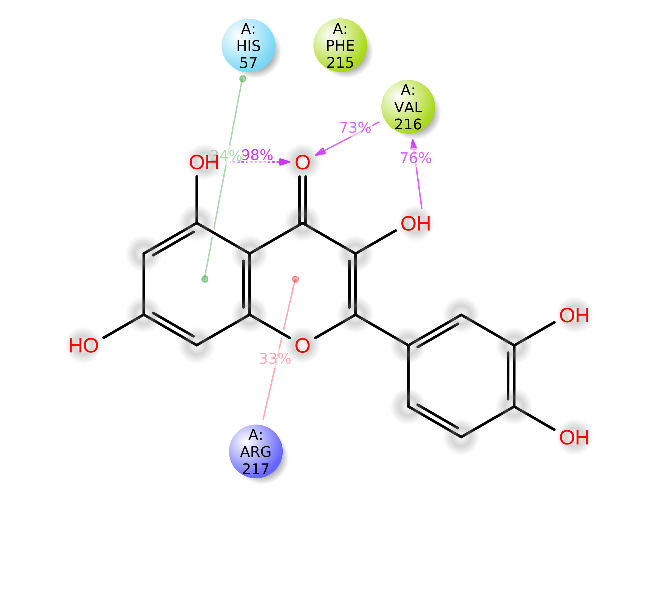


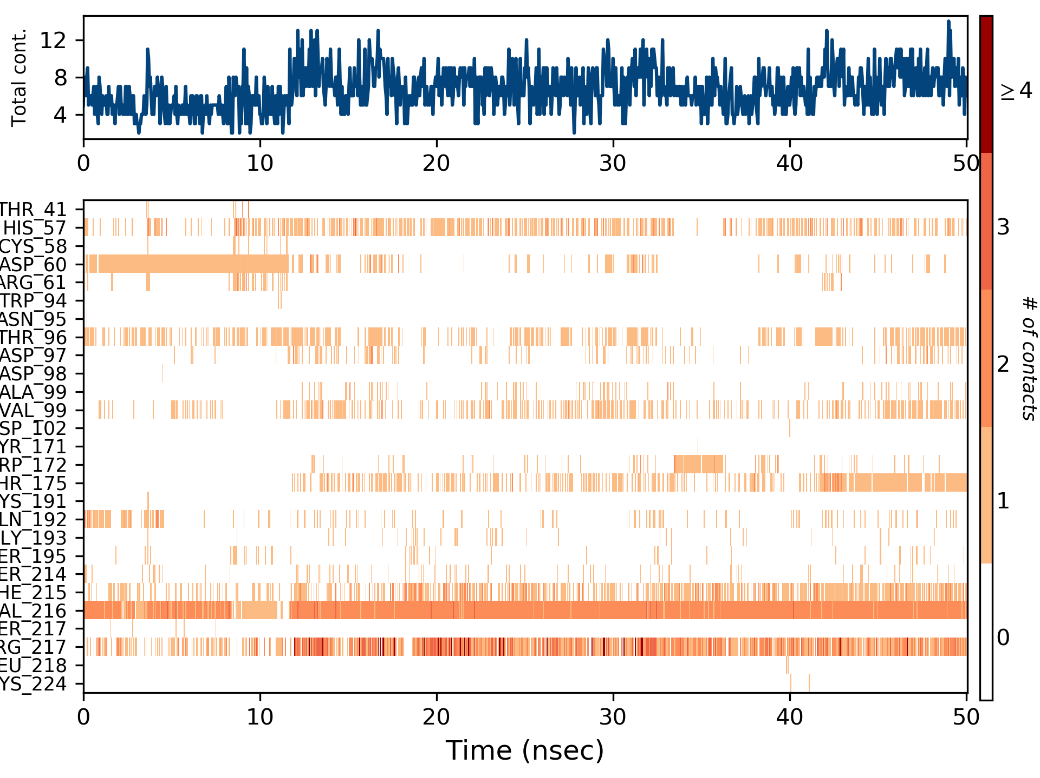


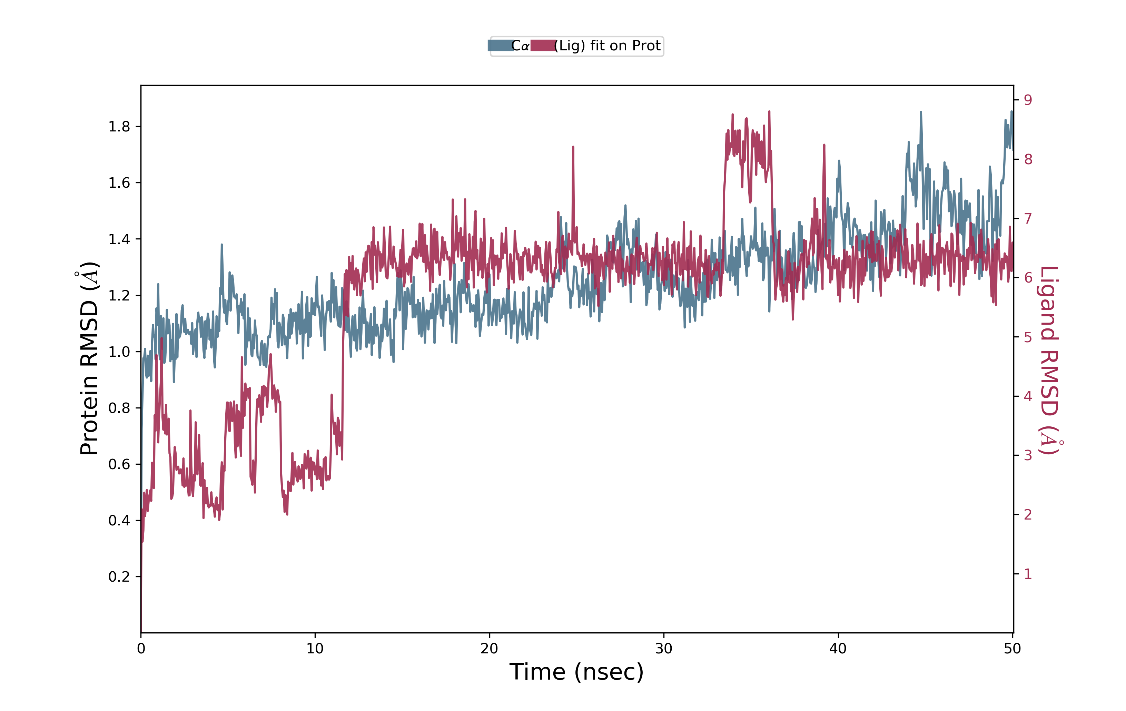


Figure s.i. 26. Molecular dynamics analysis of quercetin.


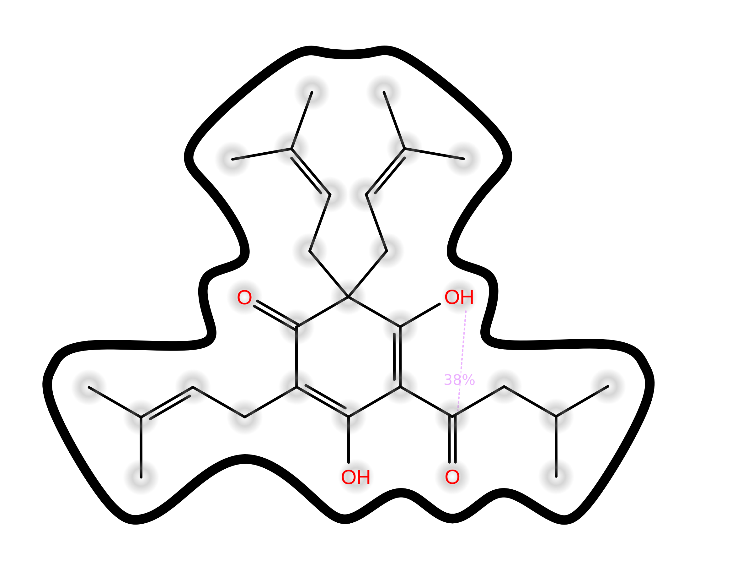


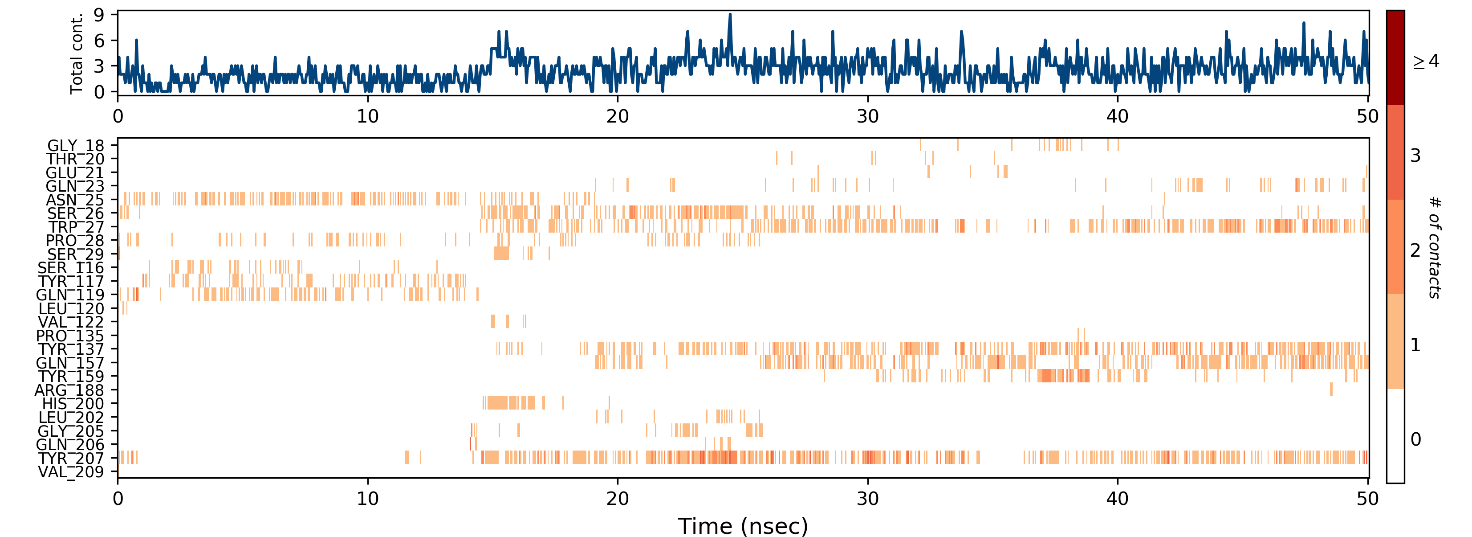


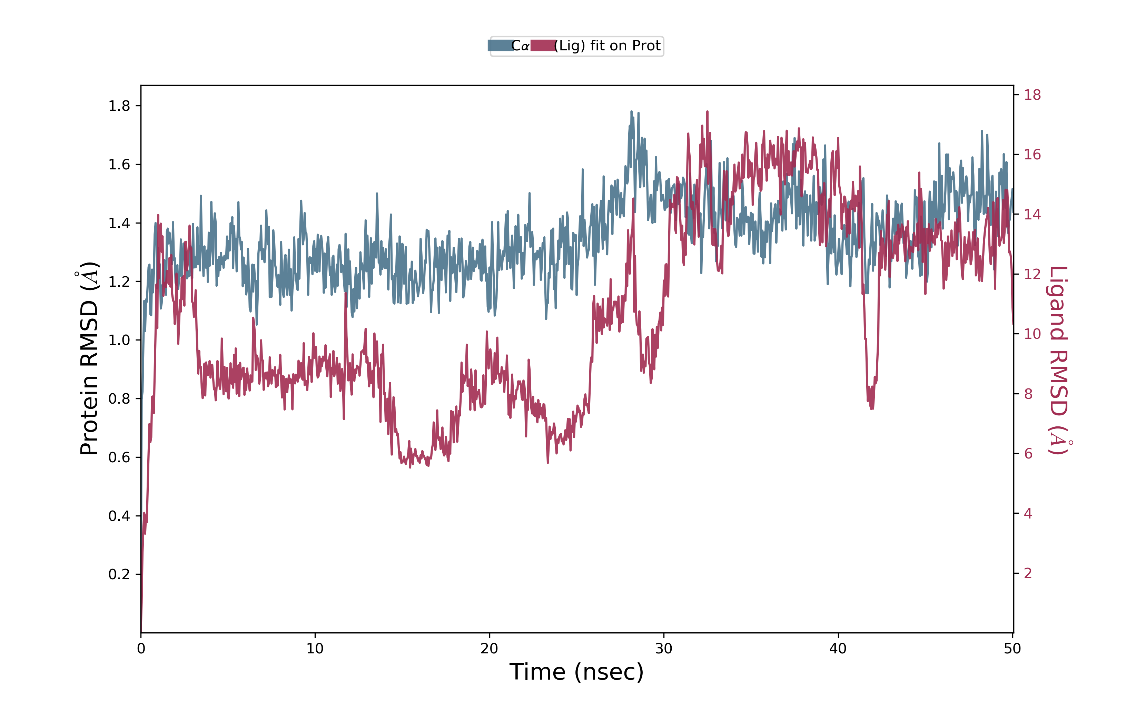


Figure s.i. 27. Molecular dynamics analysis of lupulone.


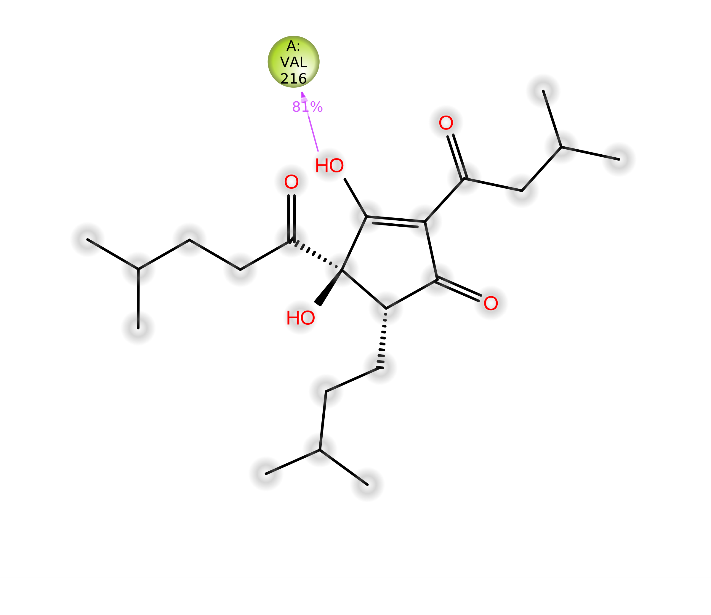


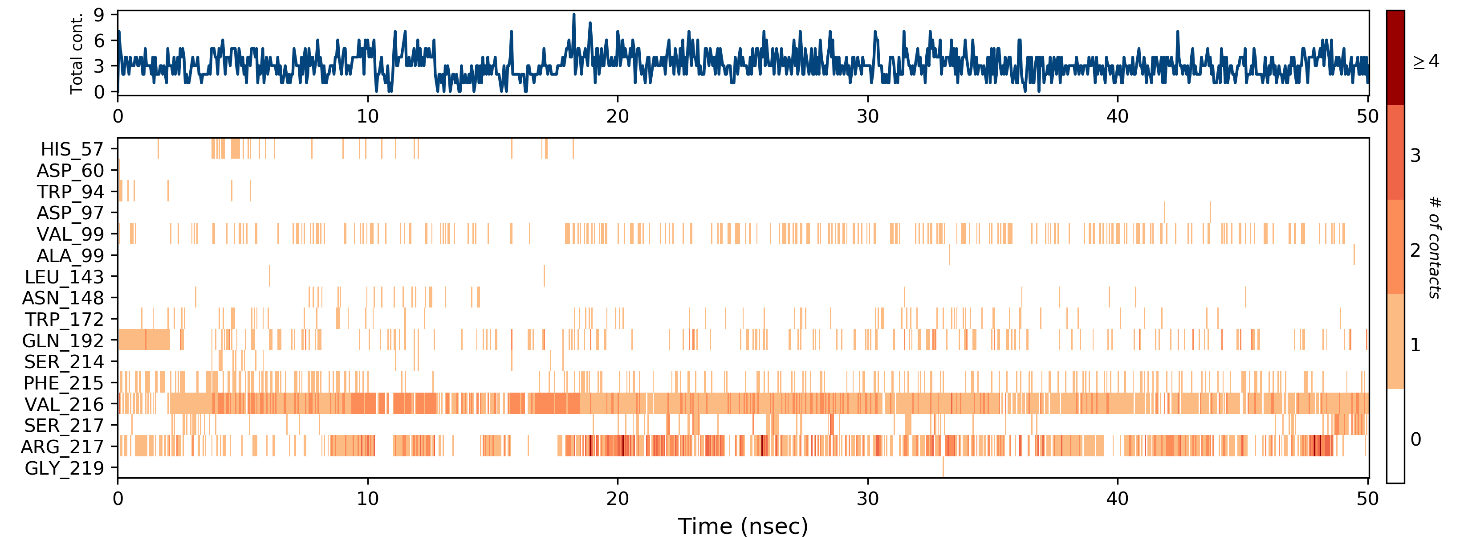


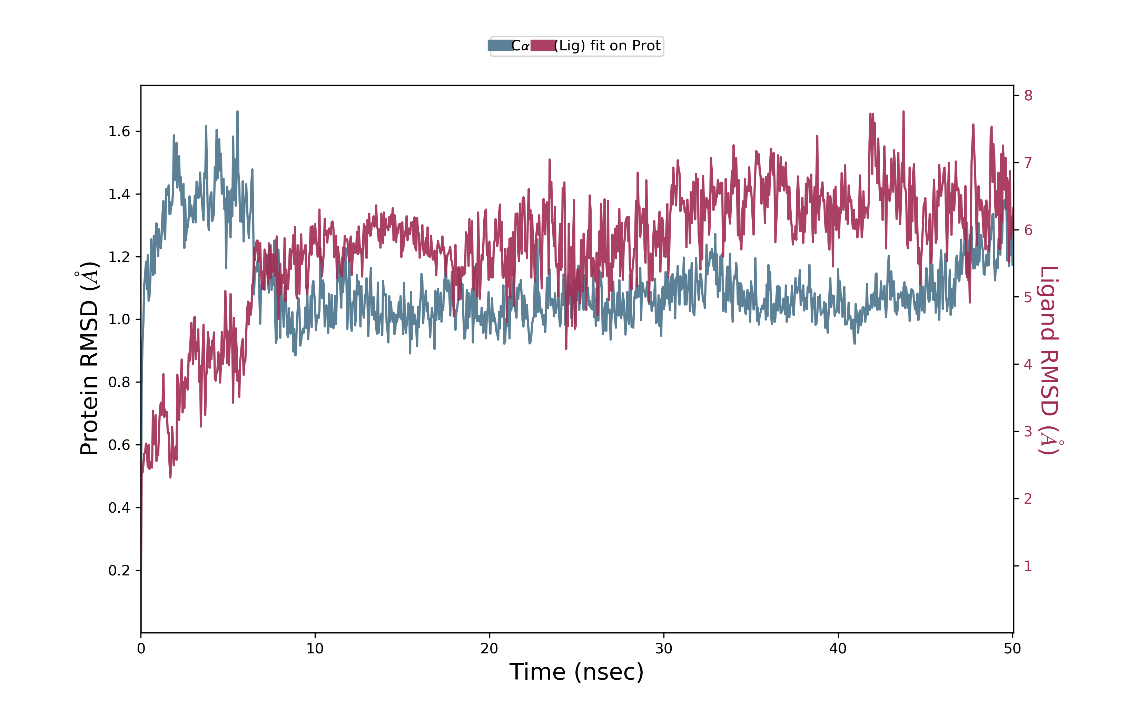


Figure s.i. 28. Molecular dynamics analysis of trans-tetrahydroisohumulone.


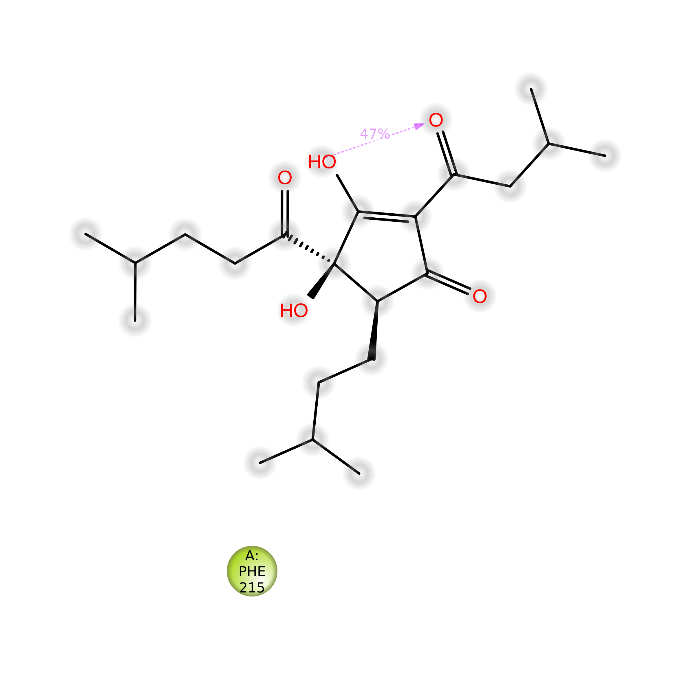

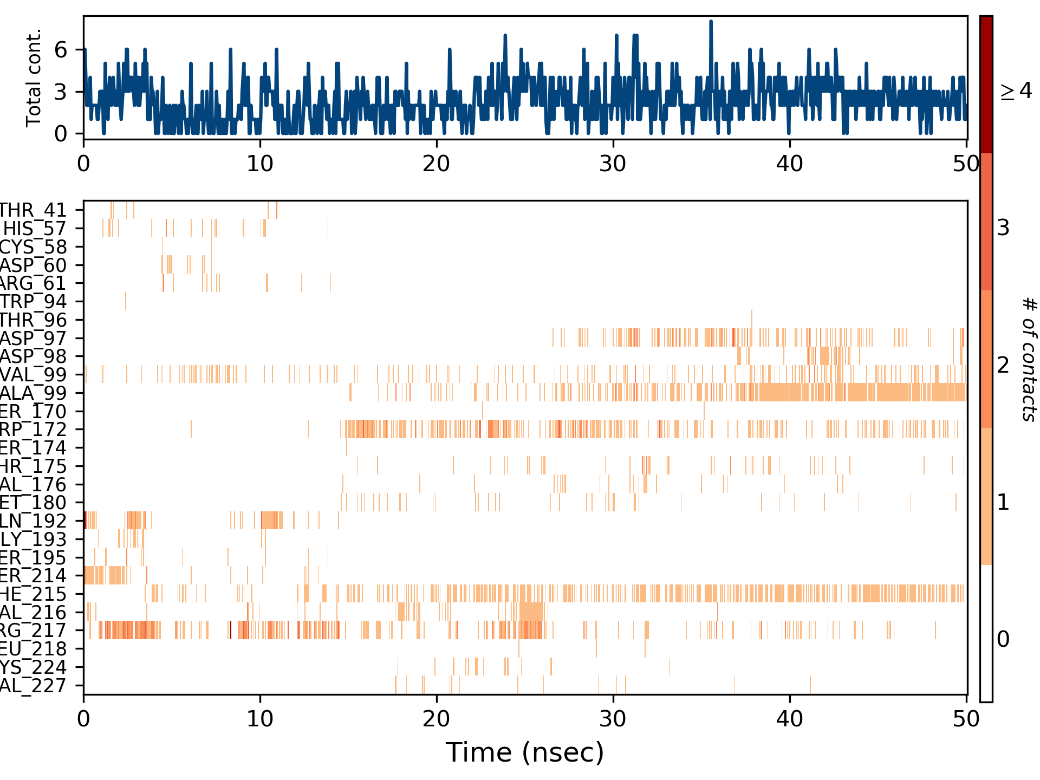


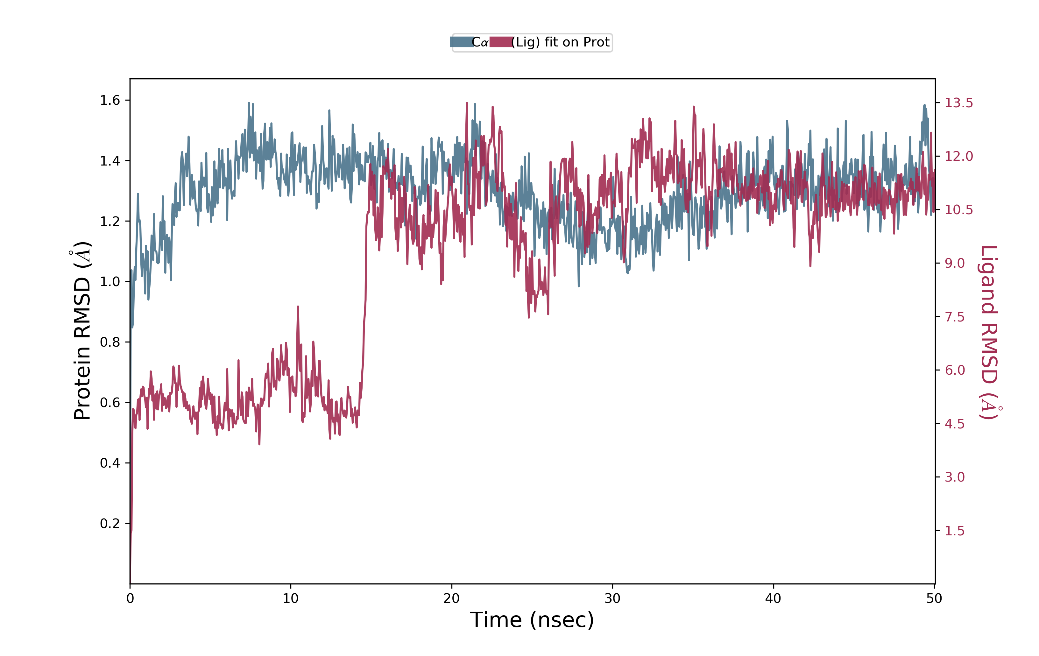


Figure s.i. 29. Molecular dynamics analysis of cis-tetrahydroisohumulone.


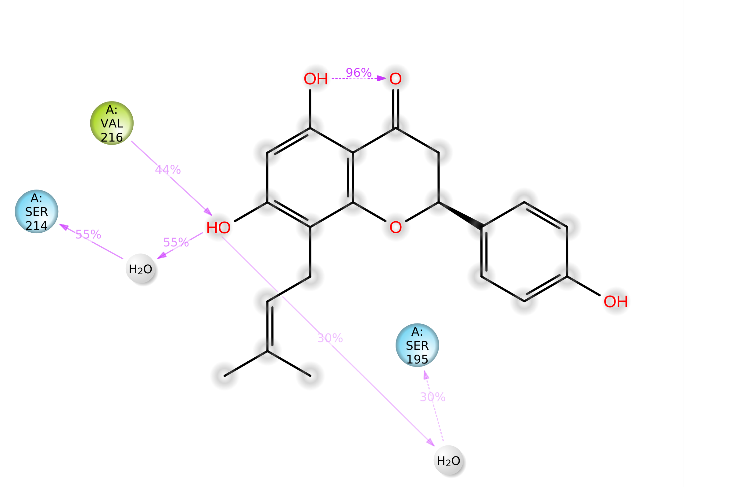


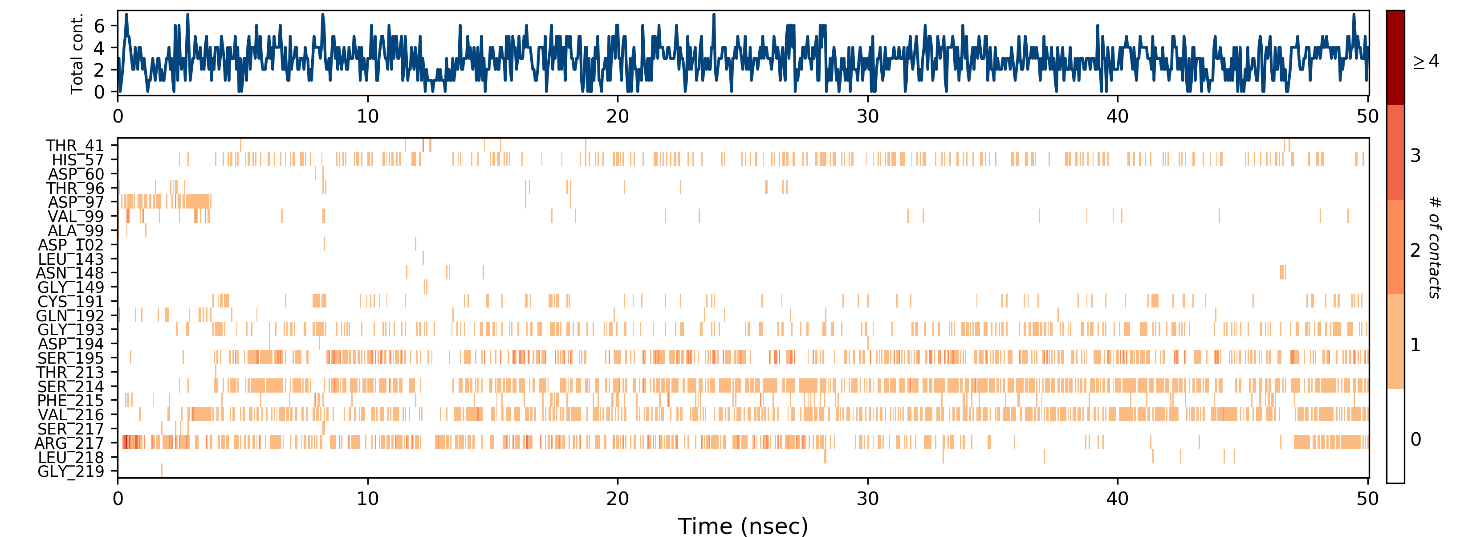


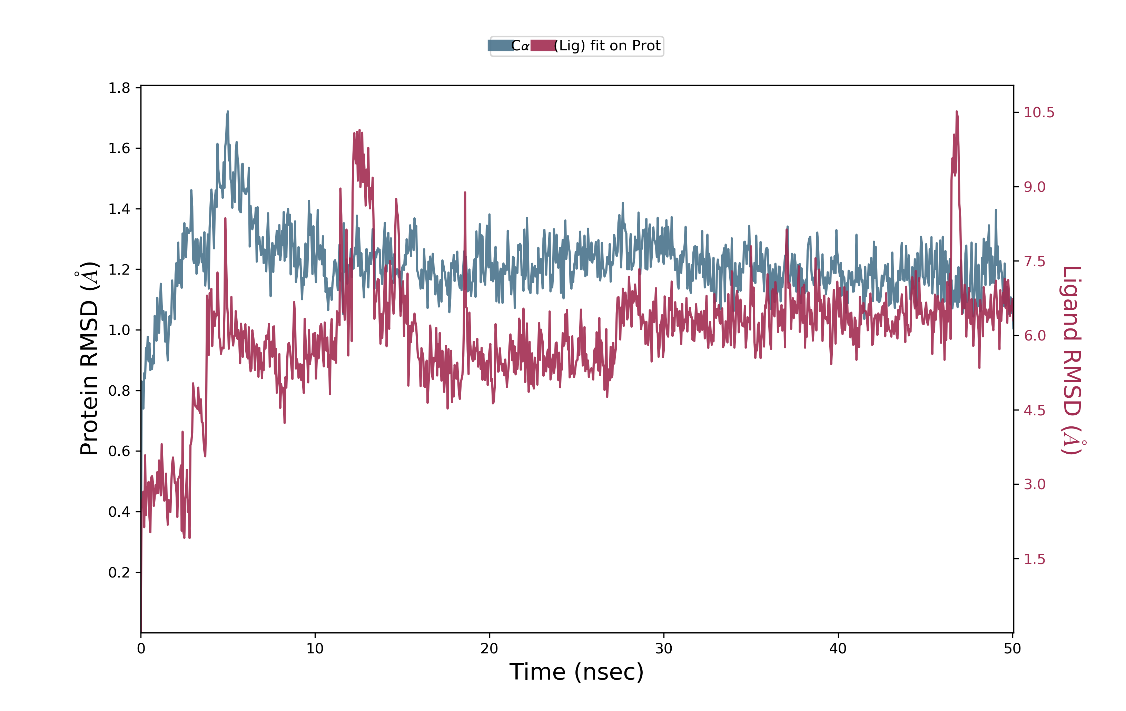


Figure s.i. 30. Molecular dynamics analysis of 8-prenylnaringenin.
